# Transauricular nerve stimulation in acute ischaemic stroke requiring mechanical thrombectomy: Protocol for a phase 2A, proof-of-concept, sham-controlled randomised trial

**DOI:** 10.1371/journal.pone.0289719

**Published:** 2023-12-22

**Authors:** Gareth L. Ackland, Tim Martin, Mareena Joseph, Priyanthi Dias, Rizwan Hameed, Ana Gutierrez del Arroyo, Russ Hewson, Tom E. F. Abbott, Oliver Spooner, Pervinder Bhogal

**Affiliations:** 1 Translational Medicine and Therapeutics, William Harvey Research Institute, Barts and The London School of Medicine and Dentistry, Queen Mary, University of London, London, United Kingdom; 2 Department of Stroke Medicine, London, Royal London Hospital, London, Barts Health NHS Trust, London, United Kingdom; 3 Department of Interventional Neuroradiology, Royal London Hospital, London, Barts Health NHS Trust, London, United Kingdom; PLoS ONE, UNITED STATES

## Abstract

**Background:**

Labile blood pressure after acute ischaemic stroke requiring mechanical thrombectomy is independently associated with poor patient outcomes.

**Objectives:**

This study protocol describes is designed to determine whether transauricular nerve stimulation, improves baroreflex sensitivity, reduces blood pressure variability in the first 24 hours after acute ischaemic stroke requiring mechanical thrombectomy.

**Design: Phase 2A, proof-of-concept, sham-controlled randomised trial:**

Methods and Analysis: 36 individuals undergoing mechanical thrombectomy for acute ischaemic stroke with established hypertension aged >18 years will be randomly allocated to receive bilateral active or sham transauricular nerve stimulation for the duration of the mechanical thrombectomy procedure (AffeX-CT/001 investigational device). The intervention will be repeated for 1h the morning following the mechanical thrombectomy. Non-invasive blood pressure will be measured ≥2h for 24h after mechanical thrombectomy. Holter electrocardiographic monitoring will be recorded during transauricular nerve stimulation. Participants, clinicians and investigators will be masked to treatment allocations. The primary outcome will be the coefficient of variation of systolic blood pressure. Secondary outcomes include additional estimates of blood pressure variability and time/frequency-domain measures of autonomic cardiac modulation An adjusted sample size of 36 patients is required to have a 90% chance of detecting, as significant at the 5% level, a difference in the coefficient of variation in systolic blood pressure of 5±4mmHg between sham and active stimulation [assuming 5% non-compliance rate in each group].

Ethics: confirmed on 16 March 2023 by HRA and Health and Care Research Wales ethics committee (reference 23/WA/0013)

**Discussion:**

This study will provide proof-of-concept data that examines whether non-invasive autonomic neuromodulation can be used to favourably modify blood pressure and autonomic control after acute ischaemic stroke requiring mechanical thrombectomy.

**Trial registration:**

**Trial registration number:** NCT05417009.

## Background

Mechanical thrombectomy has revolutionised the management of ischaemic stroke [1], but more than 50% of patients do not regain functional independence [2]. Loss of autonomic variability is strongly associated with adverse outcomes after ischaemic stroke [3,4]. Higher systolic BPV and impaired baroreflex sensitivity are independently associated with an unfavourable outcome after stroke independently of absolute blood pressure [5,6]. Impaired cardiovascular control adds prognostic value to the National Institutes of Health Stroke Scale [4].

Autonomic neuromodulation by vagus nerve stimulation paired with rehabilitation improves recovery after stroke [7]. Earlier autonomic neuromodulation during the acute phase of ischaemic stroke has not been tested, even though restoring baroreflex control and vagal activity may reduce blood pressure variability and potentially limit further cardiovascular complications [8–10]. Activation of parasympathetic innervation of blood vessels may also increase cerebral blood flow [11]. Experimental vagal nerve stimulation in rats inhibits ischemia-induced immune activation in cortical tissue [12] and dampens systemic inflammation [13]. Restoring autonomic control may limit immunosuppression and reduce post-stroke infections, which affect≈30% of patients, delay recovery and accelerate death [14].

Surgically implanted devices for autonomic neuromodulation, such as vagus nerve stimulators, are impractical in the context of acute stroke. A potential alternative is using non-invasive peripheral neuromodulation devices to attempt to restore autonomic control [15,16]. Bilateral transcutaneous aurciular stimulation is a simple, safe, hand-held, low-cost approach to increase vagal activity and baroreflex sensitivity through non-invasive, painless stimulation of nerves located in the outer ear [17]. We have demonstrated that baroreflex sensitivity can be increased at the bedside by bilateral transcutaneous aurciular stimulation following acute trauma, where the development of baroreflex dysfunction also occurs rapidly after injury [15]. Here, we describe the protocol for a randomised sham-controlled device trial, hypothesizing that bilateral transauricular nerve stimulation lowers blood pressure variability in patients presenting with acute ischaemic stroke through increasing cardiac vagal autonomic tone.

## Methods

### Study design

**Fig 1:**
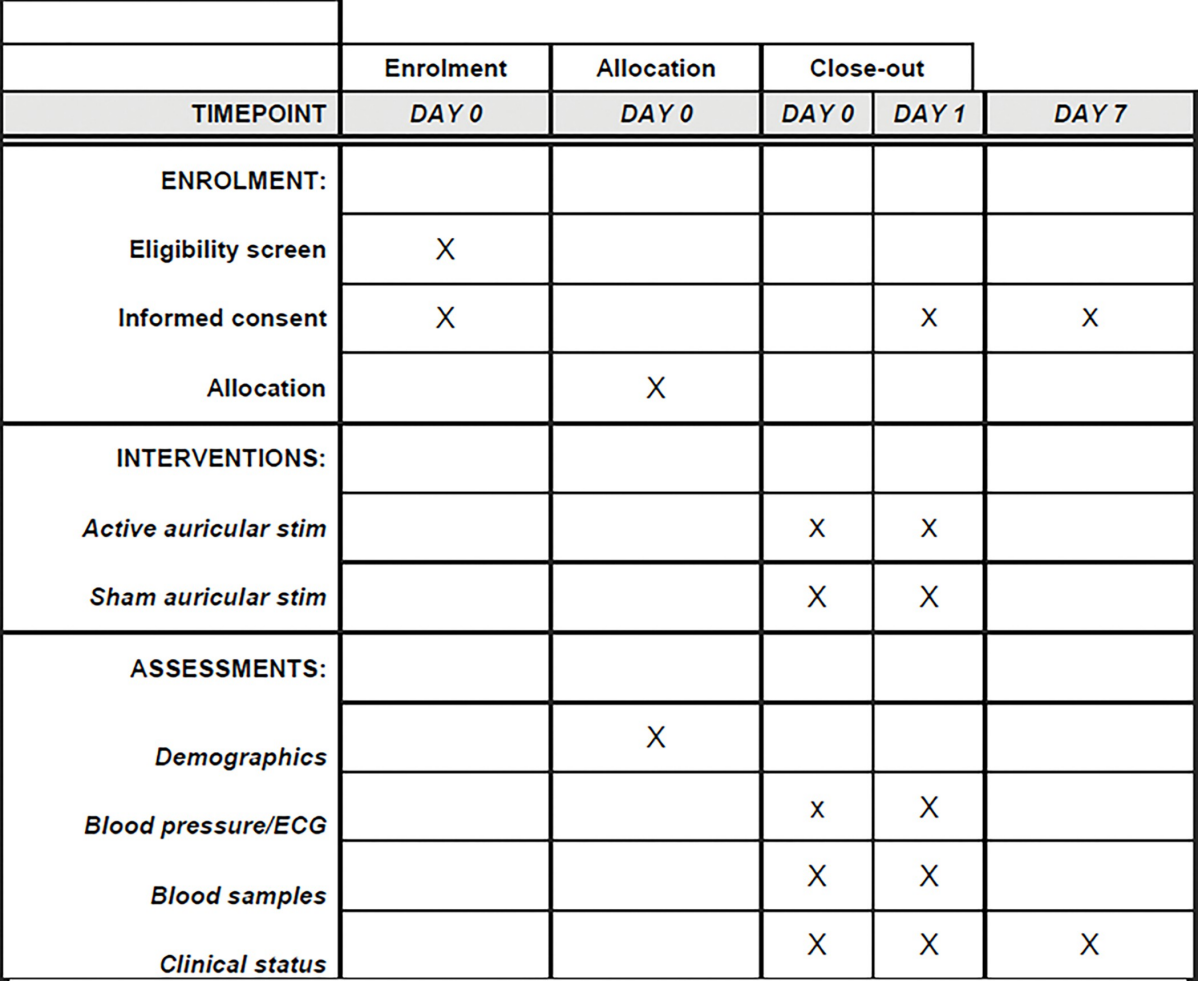
SPIRIT schedule.

This is a single centre, triple-blind, open crossover, randomised sham-controlled trial (Fig 1) undertaken at Royal London Hospital, Barts Health NHS Trust, London, United Kingdom.

Ethical permission was granted on 16 March 2023 by HRA and Health and Care Research Wales ethics committee (reference 23/WA/0013). The trial was registered at NCT05417009 Authors will have access to information that could identify individual participants during or after data collection. The completed SPIRIT schedule is shown in Fig 1.

### Inclusion criteria

- age>18 years;
- undergoing mechanical thrombectomy for acute ischaemic stroke;
- established hypertension and/or hypertensive on admission (defined as systolic BP >140mmHg; diastolic BP >90mmHg).

### Exclusion criteria

- participation in a trial exploring similar biological mechanism; previous enrolment;
- anatomical contraindication (local ear abnormalities);
- pregnancy.

#### Ethics

Screening is conducted by the interventional radiology team, who alert the research team to patients accepted for an imminent thrombectomy. Since patients presenting with acute ischaemic stroke are frequently unable to consent for themselves due to neurological impairment, sedation or delirium, a professional representative will confirm assent for participation. Subsequent to this, where patients lack capacity, a patient representative will be approached for their written consent, documented in the patient notes with a copy given to the participant and/or relative.

### Randomization

Each CE-marked device (Totally TENS, Well-Life Healthcare Ltd^TM^) used for auricular nerve stimulation is sequentially randomised by the manufacturer to deliver either sham or active stimulation. For sham stimulation, a break in the ear leads made at time of manufacture ensures that no current can be delivered. Block randomisation (blocks of 4) will be undertaken (STATA).

### Intervention

**Fig 2:**
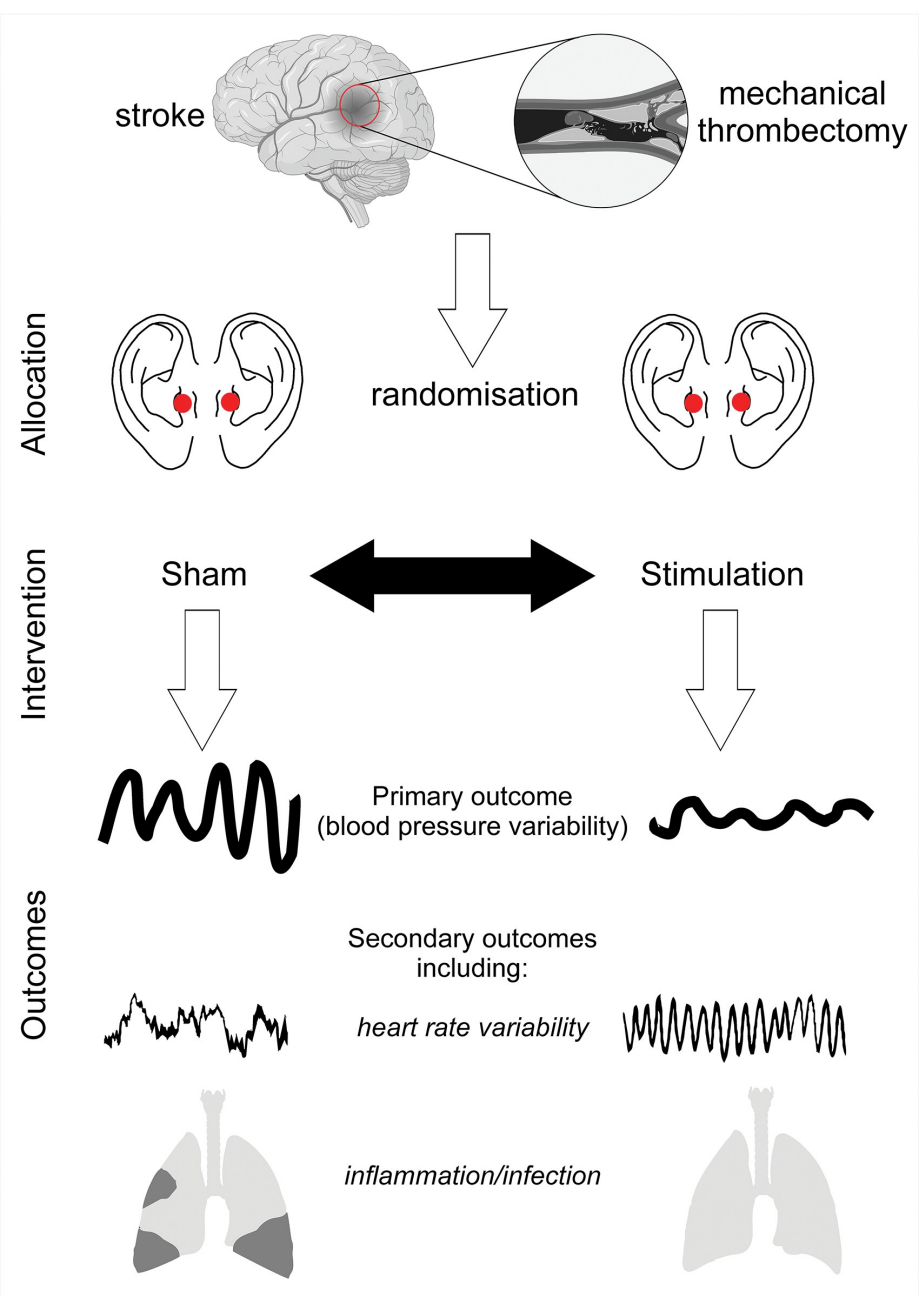
Study intervention and hypothesised outcomes. Cartoon images for stroke, thrombectomy, lungs generated using Biorender [18].

Immediately after arrival in the interventional radiology suite, continuous ECG is recorded on a Holter monitor (Spacelabs, Hertford UK). Blood pressure will be measured at least every 2h over the first 24h after mechanical thrombectomy. Once sedation or general anaesthesia are induced (according to the clinical indication determined by the attending anesthesiologists), auricular nerve stimulation will be delivered by placing two conductive clips securely on both the left and right tragus areas of the outer ear, attached to the CE-marked device. Each device is pre-programmed with parameters set at 3mA current, pulse width 200μs and frequency 30Hz, as indicated by our previous study and through systematic review [17]. (Fig 2). All devices are calibrated by oscilloscope. The leads are removed from the ears at the end of the mechanical thrombectomy (defined as removal of femoral arterial sheath). The investigator will remain with the patient to ensure compliance and adherence to the protocol. The intervention will be repeated the morning following the mechanical thrombectomy procedure for 1 hour (provided the patient has not already been discharged or repatriated to their referring centre).

### Outcome assessments

All outcome assessments are masked to treatment allocation until the final analysis.

### Primary outcome

The primary outcome will be the coefficient of variation of systolic blood pressure over the first 24h from start of mechanical thrombectomy.

### Secondary outcomes

Blood pressure variability in the first 24h after mechanical thrombectomy (standard deviation, average real variability, successive variation and residual standard deviation).

1. BPV formulae are provided in supplementary data. These measures take into account the distribution and linear trends of BP in an effort to reduce the dependency of BPV on mean BP level.
2. Autonomic cardiac modulation in the first 24h after mechanical thrombectomy (Holter monitoring).
3. Neurological recovery will be reported by recording serial NIH Stroke Scale (NIHSS) scores, an FDA-approved primary outcome measure for stroke in early phase II trials before and 24h after mechanical thrombectomy [19].

### Exploratory objectives

Biomarkers for risk of infection (whole blood leukocyte transcriptomic changes after incubation at 37°C with sterile saline or 100ng/ml lipopolysaccharide for 4h) and myocardial injury (high-sensitivity troponin) will also be quantified (Fig 2).

### Trial monitoring

An independent Trial Monitoring Committee comprising two neurologists, an internal medicine specialist, neuroanaesthetist and a clinical neuroscientist will review the RCT on alternate months. The trial steering committee is responsible for recommendations to the trial management group regarding stopping or continuing the trial. If there are adverse events of concern that may involve the use of the device, a subgroup led the Trial steering committee chair will launch a study review.

### Sample size estimation

We estimated the sample size from data reported from the secondary analysis of the BEST study, which examined blood pressure variability and neurologic outcome after endovascular thrombectomy [6]. An adjusted sample size of 36 patients is required to have a 90% chance of detecting, as significant at the 5% level, a decrease in the coefficient of variation in systolic blood pressure from 15±4mmHg in the sham group to 10±4mmHg in the VAN group [assuming 5% non-compliance rate in each group].

### Bias and blinding

Patients, clinicians and investigators are blinded to the device allocation throughout data collection and analysis. For active stimulation, the current is typically imperceptible at 3mA [15]. If patients complain or are irritated by the device leads, the current will be reduced by 1mA until the discomfort is no longer reported. Patients, clinicians and investigators are also masked to the Holter data, which will be analysed offline by an independent investigator not involved with patient recruitment/device application who is also masked to the treatment allocations.

### Data collection

Continuous ECG is recorded by Holter monitor (Spacelabs, Hertford UK). Blood pressure will be measured at least every 2h over the first 24h after mechanical thrombectomy. Plasma will be stored at -80°C having been obtained after both intervention periods for exploratory analyses of biomarkers related to autonomic activation. These data will be collected by a member of the research team not involved with administering the interventions, and analysed by an independent member of the research team–both will be masked to the treatment allocations. Data will be entered electronically on a custom-designed database.

### Statistical analysis

**Fig 3:**
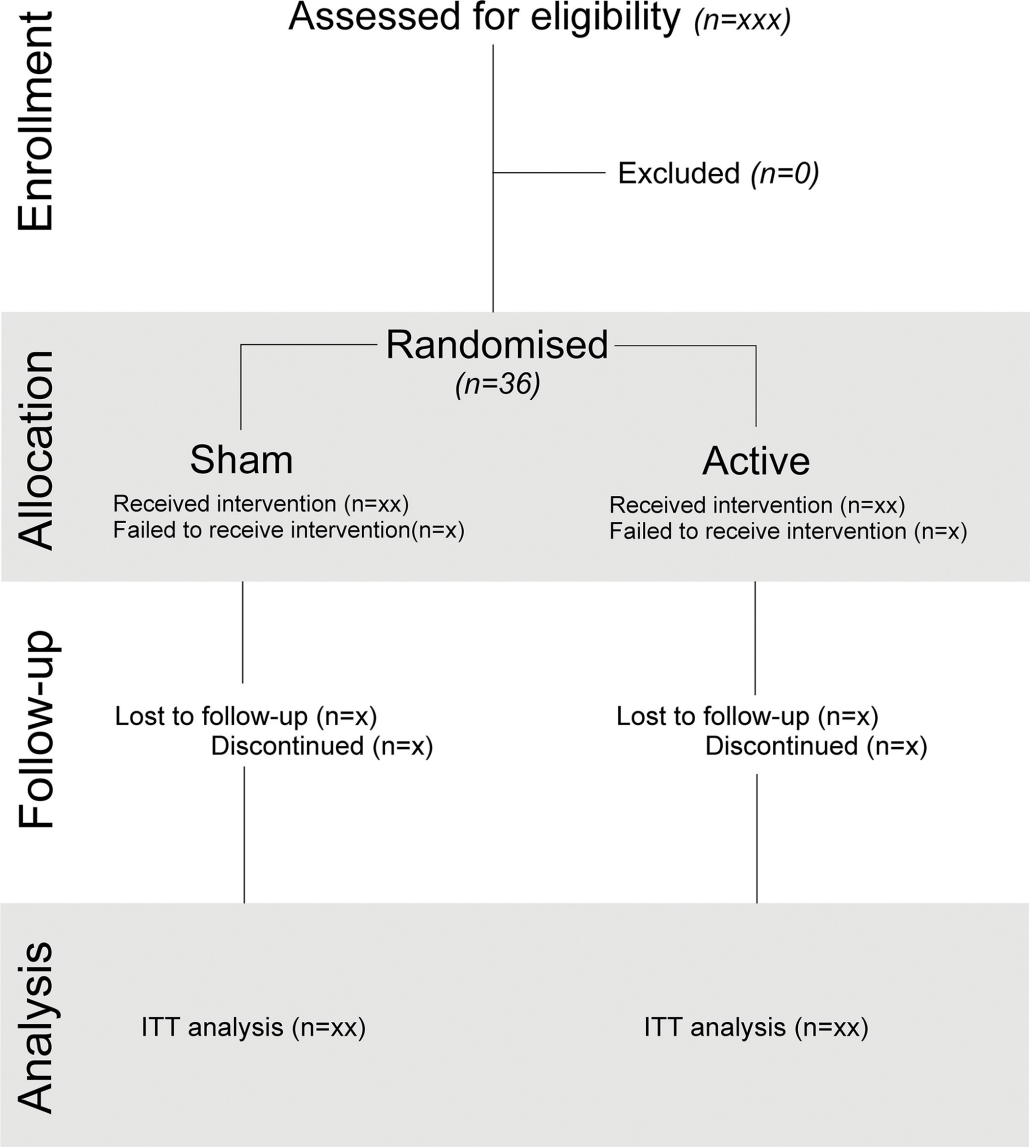
CONSORT diagram.

Analyses will be conducted using the intention-to-treat principle, where all participants with a recorded outcome are analysed according to the treatment group to which they were randomised (Fig 3). Baseline participant characteristics will be presented and stratified according to treatment allocation. The primary outcome will be analysed using repeat-measures analysis of variance. For secondary outcomes, heart rate variability data, troponin and transcirptomics will also be compared in each group using repeat-measures analysis of variance. The significance level will be set at 0.05. A full statistical analysis plan will be developed prior to analysis and the end of the study, published online at https://www.qmul.ac.uk/ccpmg/sops—saps/statistical-analysis-plans-saps.

### Trial management and data monitoring

The Sponsor organization is Queen Mary University of London (QMUL). Daily trial management will be coordinated by a trial management group consisting of the Chief Investigator and his/her support staff. An independent study committee will oversee the trial, including assessing the safety of the intervention, reviewing relevant new external evidence, and monitoring the overall conduct of the trial. This committee consists of an independent clinical trialist, lay representative and an independent Chair.

### Adverse events

Prespecified and any other adverse events directly related to the trial intervention will be recorded (supplementary material).

### Auditing

The Sponsor will have oversight of the trial conduct. The trial team ensure compliance with the requirements of Good Clinical Practice including data quality control and safety reporting.

### Dissemination plans

This is an investigator-led study sponsored by the CI’s substantive employer, QMUL. The data collected will not be used to license/register any pharmaceuticals. Data arising from the research will be made available to the scientific community in a timely and responsible manner. An appointed lay representative will co-author a detailed scientific report, which will be submitted to a widely accessible scientific journal. Authorship of the final manuscript(s), interim publications, or abstracts will be decided according to active participation in the design, accrual of eligible participants and statistical analysis. Contributing/participating investigators will be acknowledged in the final manuscript.

## Discussion

Current treatments for ischemic stroke mainly focus on revascularization or reperfusion of cerebral blood flow to the infarcted tissue [20]. Despite advances with mechanical thrombectomy, reperfusion injury may exacerbate ischemic injury in patients with stroke. Experimental models suggest that autonomic neuromodulation, chiefly involving direct stimulation of the vagus nerve, may improve cognitive outcomes and minimise neuronal deficits [21].

Autonomic neuromodulation by vagus nerve stimulation paired with rehabilitation improves recovery after stroke [7]. Earlier autonomic neuromodulation during the acute phase of ischaemic stroke has not been tested, even though restoring baroreflex control and vagal activity may reduce blood pressure variability and potentially limit further cardiovascular complications [8–10]. Activation of parasympathetic innervation of blood vessels may also increase cerebral blood flow [11]. Experimental vagal nerve stimulation in rats inhibits ischemia-induced immune activation in cortical tissue [12] and dampens systemic inflammation [13]. Restoring autonomic control may limit immunosuppression and reduce post-stroke infections, which affect≈30% of patients, delay recovery and accelerate death [14]. The concept of non-invasive approaches to improve blood pressure control during the acute phase of ischaemic stroke by enhancing efferent vagal activity is very attractive [22,23]. However, whether transauricular nerve stimulation results in vagal activation specifically remains uncertain, with variable components of heart rate variability reportedly altered by active stimulation in volunteers [24]. Very few clinical studies have actively sought to measure cardiac autonomic modulation at the same time as the primary outcome has been recorded [17]. Although the assessment of autonomic function is particularly challenging during acute tissue injury in patients, we found that only active transauricular nerve stimulation altered HRV measures indicative of increased baroreflex sensitivity [15]. These data were consistent with our systematic review that identified stimulation parameters that alter heart rate variability [17]. A 60 minute period of daily stimulation has not been associated with any major adverse events [17]. Moreover, our data were consistent with numerous previous reports detailing that hypertensive individuals have blunted baroreflex sensitivity. which is positively related to heart rate variability and inversely related to blood pressure variability. Baroreflex sensitivity is also inversely correlated with ambulatory blood pressure in healthy non-hypertensive humans [25].

To reduce bias, which is common in studies of non-invasive autonomic neuromodulation [17], we will utilise a triple-blind, randomised controlled design. The use of a sham stimulation device is critical to minimise participant and investigator biases. Far more invasive studies have successfully recruited patients into trials where sham procedures served as the key control in cardiac [26] and noncardiac orthopaedic surgery [27] Our prospective randomised study will ensure that there are a high number of BP measurements (37) for the 24 hour exposure period, which increase the accuracy of estimating BPV. Although we will not be able to control concomitant medical interventions that may alter BP (such as mode of anaesthesia/sedation), multi-level statistical analytic techniques will account for these potential confounding factors. In contrast to observational studies such as BEST, the intervention can directly provide further insight into whether BPV is a modifiable factor rather than merely being an epiphenomenon.

In summary, this randomised sham-controlled trial will assess whether non-invasive autonomic neuromodulation may reduce blood pressure variability and favourably alter autonomic control. This non-pharmacologic approach may be broadly generalisable to help improve cardiovascular health after acute ischaemic stroke.

## Supporting information

S1 Checklist SPIRIT 2013 checklist: Recommended items to address in a clinical trial protocol and related documents*. (DOC)

S1 File (PDF)

## References

[1] Bhogal P, Andersson T, Maus V, Mpotsaris A, Yeo L. Mechanical Thrombectomy-A Brief Review of a Revolutionary new Treatment for Thromboembolic Stroke. Clin Neuroradiol. 2018;28(3):313–26. doi: 10.1007/s00062-018-0692-2 . 29744519

[2] Goyal M, Menon BK, van Zwam WH, Dippel DW, Mitchell PJ, Demchuk AM, et al. Endovascular thrombectomy after large-vessel ischaemic stroke: a meta-analysis of individual patient data from five randomised trials. Lancet. 2016;387(10029):1723–31. doi: 10.1016/S0140-6736(16)00163-X . 26898852

[3] Jafari M, Desai A, Damani R. Blood pressure management after mechanical thrombectomy in stroke patients. J Neurol Sci. 2020;418:117140. doi: 10.1016/j.jns.2020.117140 . 32961389

[4] Tang S, Xiong L, Fan Y, Mok VCT, Wong KS, Leung TW. Stroke Outcome Prediction by Blood Pressure Variability, Heart Rate Variability, and Baroreflex Sensitivity. Stroke. 2020;51(4):1317–20. doi: 10.1161/STROKEAHA.119.027981 . 31964286

[5] Sykora M, Diedler J, Turcani P, Hacke W, Steiner T. Baroreflex: a new therapeutic target in human stroke? Stroke. 2009;40(12):e678–82. doi: 10.1161/STROKEAHA.109.565838 . 19834010

[6] Mistry EA, Mehta T, Mistry A, Arora N, Starosciak AK, De Los Rios La Rosa F, et al. Blood Pressure Variability and Neurologic Outcome After Endovascular Thrombectomy: A Secondary Analysis of the BEST Study. Stroke. 2020;51(2):511–8. doi: 10.1161/STROKEAHA.119.027549 ; PubMed Central PMCID: PMC8010595. 31813361 PMC8010595

[7] Dawson J, Liu CY, Francisco GE, Cramer SC, Wolf SL, Dixit A, et al. Vagus nerve stimulation paired with rehabilitation for upper limb motor function after ischaemic stroke (VNS-REHAB): a randomised, blinded, pivotal, device trial. Lancet. 2021;397(10284):1545–53. doi: 10.1016/S0140-6736(21)00475-X ; PubMed Central PMCID: PMC8862193. 33894832 PMC8862193

[8] Maier B, Turc G, Taylor G, Blanc R, Obadia M, Smajda S, et al. Prognostic Significance of Pulse Pressure Variability During Mechanical Thrombectomy in Acute Ischemic Stroke Patients. J Am Heart Assoc. 2018;7(18):e009378. doi: 10.1161/JAHA.118.009378 ; PubMed Central PMCID: PMC6222945. 30371208 PMC6222945

[9] Stavrakis S, Humphrey MB, Scherlag BJ, Hu Y, Jackman WM, Nakagawa H, et al. Low-level transcutaneous electrical vagus nerve stimulation suppresses atrial fibrillation. J Am Coll Cardiol. 2015;65(9):867–75. doi: 10.1016/j.jacc.2014.12.026 ; PubMed Central PMCID: PMC4352201. 25744003 PMC4352201

[10] Mastitskaya S, Marina N, Gourine A, Gilbey MP, Spyer KM, Teschemacher AG, et al. Cardioprotection evoked by remote ischaemic preconditioning is critically dependent on the activity of vagal pre-ganglionic neurones. Cardiovasc Res. 2012;95(4):487–94. doi: 10.1093/cvr/cvs212 ; PubMed Central PMCID: PMC3422080. 22739118 PMC3422080

[11] Roloff EV, Tomiak-Baquero AM, Kasparov S, Paton JF. Parasympathetic innervation of vertebrobasilar arteries: is this a potential clinical target? J Physiol. 2016;594(22):6463–85. doi: 10.1113/JP272450 ; PubMed Central PMCID: PMC5108906. 27357059 PMC5108906

[12] Ay I, Nasser R, Simon B, Ay H. Transcutaneous Cervical Vagus Nerve Stimulation Ameliorates Acute Ischemic Injury in Rats. Brain Stimul. 2016;9(2):166–73. doi: 10.1016/j.brs.2015.11.008 ; PubMed Central PMCID: PMC4789082. 26723020 PMC4789082

[13] Chavan SS, Pavlov VA, Tracey KJ. Mechanisms and Therapeutic Relevance of Neuro-immune Communication. Immunity. 2017;46(6):927–42. doi: 10.1016/j.immuni.2017.06.008 ; PubMed Central PMCID: PMC5578398. 28636960 PMC5578398

[14] Westendorp WF, Nederkoorn PJ, Vermeij JD, Dijkgraaf MG, van de Beek D. Post-stroke infection: a systematic review and meta-analysis. BMC Neurol. 2011;11:110. doi: 10.1186/1471-2377-11-110 ; PubMed Central PMCID: PMC3185266. 21933425 PMC3185266

[15] Patel ABU, Bibawy P, Althonayan JIM, Majeed Z, Gan WL, Abbott TEF, et al. Effect of transauricular nerve stimulation on perioperative pain: a single-blind, analyser-masked, randomised controlled trial. Br J Anaesth. 2023;130(4):468–76. doi: 10.1016/j.bja.2022.12.025 ; PubMed Central PMCID: PMC10080471. 36822987 PMC10080471

[16] Clancy JA, Mary DA, Witte KK, Greenwood JP, Deuchars SA, Deuchars J. Non-invasive vagus nerve stimulation in healthy humans reduces sympathetic nerve activity. Brain Stimul. 2014;7(6):871–7. doi: 10.1016/j.brs.2014.07.031 . 25164906

[17] Patel ABU, Weber V, Gourine AV, Ackland GL. The potential for autonomic neuromodulation to reduce perioperative complications and pain: a systematic review and meta-analysis. Br J Anaesth. 2022;128(1):135–49. doi: 10.1016/j.bja.2021.08.037 . 34801224 PMC8787777

[18] Adapted from “Lung” template, biorender.com [Internet]. 2023 [cited 27/07/2023]. Available from: https://app.biorender.com/biorender-templates.

[19] Chalos V, van der Ende NAM, Lingsma HF, Mulder M, Venema E, Dijkland SA, et al. National Institutes of Health Stroke Scale: An Alternative Primary Outcome Measure for Trials of Acute Treatment for Ischemic Stroke. Stroke. 2020;51(1):282–90. doi: 10.1161/STROKEAHA.119.026791 ; PubMed Central PMCID: PMC6924951. 31795895 PMC6924951

[20] Fayad P. Improved Prospects for Thrombectomy in Large Ischemic Stroke. N Engl J Med. 2023;388(14):1326–8. doi: 10.1056/NEJMe2300193 . 36762847

[21] Keser Z, Feng W. Vagus Nerve Stimulation for Stroke Motor Recovery-What Is Next? Transl Stroke Res. 2022. doi: 10.1007/s12975-022-01041-4 . 35653016

[22] Cai PY, Bodhit A, Derequito R, Ansari S, Abukhalil F, Thenkabail S, et al. Vagus nerve stimulation in ischemic stroke: old wine in a new bottle. Front Neurol. 2014;5:107. doi: 10.3389/fneur.2014.00107 ; PubMed Central PMCID: PMC4067569. 25009531 PMC4067569

[23] Jiang Y, Li L, Ma J, Zhang L, Niu F, Feng T, et al. Auricular vagus nerve stimulation promotes functional recovery and enhances the post-ischemic angiogenic response in an ischemia/reperfusion rat model. Neurochem Int. 2016;97:73–82. doi: 10.1016/j.neuint.2016.02.009 . 26964767

[24] Wolf V, Kuhnel A, Teckentrup V, Koenig J, Kroemer NB. Does transcutaneous auricular vagus nerve stimulation affect vagally mediated heart rate variability? A living and interactive Bayesian meta-analysis. Psychophysiology. 2021;58(11):e13933. doi: 10.1111/psyp.13933 . 34473846

[25] Hesse C, Charkoudian N, Liu Z, Joyner MJ, Eisenach JH. Baroreflex sensitivity inversely correlates with ambulatory blood pressure in healthy normotensive humans. Hypertension. 2007;50(1):41–6. doi: 10.1161/HYPERTENSIONAHA.107.090308 . 17502489

[26] Uhe T, Beimel S, Langhammer R, Stegmann T, Hindricks G, Laufs U, et al. Patients’ attitude towards a sham-controlled trial on pulmonary vein isolation in atrial fibrillation. Clin Res Cardiol. 2022;111(1):114–23. doi: 10.1007/s00392-021-01959-z ; PubMed Central PMCID: PMC8766391. 34709451 PMC8766391

[27] Sihvonen R, Paavola M, Malmivaara A, Itala A, Joukainen A, Nurmi H, et al. Arthroscopic partial meniscectomy versus sham surgery for a degenerative meniscal tear. N Engl J Med. 2013;369(26):2515–24. doi: 10.1056/NEJMoa1305189 . 24369076

