# Supplementary material for: Transauricular nerve stimulation in acute ischaemic stroke requiring mechanical thrombectomy: Protocol for a phase 2A, proof-of-concept, sham-controlled randomised trial
Source: PLoS One. 2023 Dec 22;18(12):e0289719. doi: 10.1371/journal.pone.0289719 (PMC10745208; doi:10.1371/journal.pone.0289719)
Supplement: S1 File — (PDF) [file pone.0289719.s002.pdf]

**Full Title:**	**V**agal **A**utonomic **N**euromodulation by transcutaneous nerve stimulation in acute ischaemic **S**troke requiring mechanical thrombectomy: a phase 2A, sham controlled randomised trial
**Short Title**	VANS
**Sponsor**	Queen Mary University of London
**Contact person**	Dr Mays Jawad<br>Governance Operations Manager<br>Joint Research Management Office<br>Dept W, Research Services<br>69-89 Mile End Road<br>London E1 4UJ<br>Phone: +44 (0) 20 7882 7275<br>Email: research.governance@qmul.ac.uk
**IRAS Number**	314067
**EDGE ID**	151667
**NCT**	NCT05417009
**Chief Investigator**	Professor Gareth Ackland<br>Translational Medicine & Therapeutics (218A)<br>Barts and The London School of Medicine and Dentistry<br>William Harvey Research Institute, QMUL<br>John Vane Science Centre, Charterhouse Square<br>London, EC1M 6BQ<br>Phone: +44 (0)20 7882 2100<br>Email: g.ackland@qmul.ac.uk

Study Contacts

Trial Manager	Dr Priyanthi Dias<br>Clinical Trial and Research Manager<br>ACCU Research Offices<br>4th Floor, Office 14<br>Royal London Hospital<br>Whitechapel, London E1 1FR<br>Tel: +44 (0)20 3594 0349
Funder(s)	INNOVATE-UK/ Afferent Medical Solutions Ltd<br>4 Willoughby Drive<br>Solihull West Midlands<br>B91 3GB<br>United Kingdom<br>Telephone 07815444305
Statistician	Tom Abbott MA PhD MRCP FRCA<br>NIHR Clinical Lecturer in Anaesthesia & Perioperative Medicine<br>William Harvey Research Institute<br>Queen Mary University of London
Device Manufacturer	Afferent Medical Solutions Ltd<br>4 Willoughby Drive<br>Solihull West Midlands<br>B91 3GB<br>United Kingdom
Committees<br>(IDMC, TSC, TMG)	**IDMC Chair:** Prof Tony Pickering, Bristol<br>(Tony.Pickering@bristol.ac.uk)<br><br>**TSC Chair:** Prof. Johannes Boltze, Univ. Warwick.<br>(johannes.boltze@warwick.ac.uk)<br><br>**TMG Chair:** Prof. Gareth Ackland
**Key CIP contributors**	Study design: Ackland<br>Conduct: Ackland, May, Dias, Bhogal<br>Data analysis and interpretation: Abbott, Ackland<br>Manuscript writing: Ackland/TMG<br>Dissemination of results: Ackland<br><br>The Sponsor or Funder does not control any final decisions regarding any of these aspects
**List of laboratories**	1. The Doctors Laboratory, 1 Mabledon Pl, London WC1H 9AX<br>2. William Harvey Research Institute, QMUL, John Vane Science Centre, Charterhouse Square, London EC1M 6BQ

Contents

1.0 Introduction 7
2.0 Trial objectives 9
3.0 Patient Evaluability and Replacement 11
4.0 Informed consent procedures 11
5.0 Participant eligibility criteria 13
6.0 Study Schedule 13
7.0 Participant, Study, and Site discontinuation 19
8.0 Laboratories and samples 19
9.0 Investigational Device 20
10.0 Legal status of Investigational Device .... 21~~22~~
11.0 Safety Reporting 28
12.0 Annual reporting .... 30~~31~~
13.0 Statistical and data analysis .... 30~~31~~
14.0 Statistical Analysis .... 31~~32~~
15.0 Data handling and record keeping 33
16.0 Confidentiality .... 34~~35~~
17.0 Monitoring, Audit, and Inspection .... 35~~36~~
18.0 Compliance .... 35~~36~~
19.0 Declaration of interests 37
20.0 Peer review .... 37~~38~~
21.0 Public and Patient Involvement (PPI) .... 37~~38~~
22.0 Indemnity .... 37~~38~~
23.0 Study committees 38
24.0 Publication and dissemination policy .... 38~~39~~
25.0 Archiving .... 39~~40~~
26.0 References .... 40~~41~~
27.0 Appendix A 42

II. Glossary of terms and abbreviations

AE	Adverse Event
ADE	Adverse Device Event
AR	Adverse Reaction
BP	Blood Pressure
APR	Annual Progress Report
DMEC	Data Monitoring and Ethics Committee
eCRF	Electronic Case Report Form
GCP	Good Clinical Practice
ISF	Investigator Site File
JRMO	Joint Research Management Office
MHRA	Medicines and Healthcare products Regulatory Agency
Participant	An individual who takes part in a clinical study
QMUL	Queen Mary University of London
REC	Research Ethics Committee
RSI	Reference Safety Information
SAE	Serious Adverse Event
SADE	Serious Adverse Device Event
SAR	Serious Adverse Reaction
SDA	Source Data Agreement
SUSAR	Suspected Unexpected Serious Adverse Reaction
TAN	Transcutaneous autonomic neuromodulation
TMF	Trial Master File
TMG	Trial Management Group
TSC	Trial Steering Committee
USADE	Unexpected Serious Device Event
WHRI	William Harvey Research Institute

III. Signature page

Chief Investigator Agreement

The study as detailed within this clinical investigation plan will be conducted in accordance with the principles of Good Clinical Practice, the UK Policy Framework for Health and Social Care Research, the Declaration of Helsinki, and the current regulatory requirements, including the Medical Device Regulations 2002 and all subsequent amendments. I delegate responsibility for the statistical analysis and oversight to a qualified statistician (see declaration below).

Chief Investigator name: Professor Gareth Ackland

Signature: _________________

Date: _________________

Statistician's Agreement

The statistical aspects of the clinical study as detailed in this clinical investigation plan are in accordance with the principles of Good Clinical Practice, the UK Policy Framework for Health and Social Care Research, the Declaration of Helsinki, and the current regulatory requirements, including the Medical Device Regulations 2002 and all subsequent amendments.

I take responsibility for ensuring the statistical work in this clinical investigation plan is accurate, and for the statistical analysis and oversight of this study.

Statistician's name: Dr Tom Abbott

Signature: _________________

Date: _________________

Principal Investigator Agreement Page

The clinical study as detailed within this clinical investigation plan, or any subsequent amendments, involves the use of an investigational medical device and will be conducted in accordance with the UK Policy Framework for Health and Social Care Research, the World Medical Association Declaration of Helsinki (1996), Principles of ISO14155 GCP, and the current regulatory requirements, as detailed in the Medical Device Regulations 2002 and any subsequent amendments of the regulations.

Principal Investigator Name: Professor Gareth Ackland

Principal Investigator Site: Barts Health NHS Trust

Signature and Date: _________________

IV. Synopsis

Full title	Autonomic modulation by transcutaneous vagal nerve stimulation in acute ischaemic stroke requiring mechanical thrombectomy: a phase IIa, sham controlled randomised trial.
Short title and / or acronym	VANS
Sponsor	Queen Mary, University of London
Device classification	Class IIa
Medical condition or disease under investigation	Acute ischaemic stroke requiring mechanical thrombectomy
Study design and methodology	Triple-blind, randomised controlled trial, single NHS -site
Planned number of participants	36
Objectives	To determine whether non-invasive transcutaneous autonomic neuromodulation results in lower blood pressure variability in the first 24 hours from the start of mechanical thrombectomy, as measured by coefficient of variation compared to the control treatment.
Inclusion and exclusion criteria	Inclusion criteria:<br>• age>18 years<br>• undergoing mechanical thrombectomy for acute ischaemic stroke<br>• established hypertension and/or hypertensive on admission (systolic BP >140mmHg; diastolic BP >80mmHg)<br>Exclusion criteria:<br>• participation in a trial exploring similar biological mechanism<br>• previous enrolment<br>• anatomical contraindication<br>• pregnancy
Investigational Device(s)	AffeX-CT/001
Treatment duration	Approximately 1 – 3 hours of treatment across two separate calendar days.
Follow up duration	Seven days after index mechanical thrombectomy
Total duration for participants	Four months
Planned Enrolment Period	Six months
End of Trial definition	When the last patient has completed their seven day follow-up

1.0 Introduction

Background

Loss of autonomic variability is strongly associated with adverse outcomes after ischaemic stroke. Removing blood clots from the brain by mechanical thrombectomy has revolutionised the management of stroke, but more than 50% of patients do not regain functional independence.(PMID:26898852) Blood pressure (BP) control is important, since low and high BP (BP variability) are strongly associated with poor patient outcomes after thrombectomy.(PMIDs:32961389;31964286) Current therapy centre son the use of anti-hypertensive medication, but these drugs do not address lability of blood pressure, often have poor adherence and can be difficult for patients after a stroke to use.

Autonomic dysfunction causes labile blood pressure. Intact autonomic function is required to control blood pressure and potentially improve recovery after stroke. Impairment of baroreflex autonomic function, due to reduced vagal activity is associated with extreme BP variability, leading to further brain injury and cardiovascular complications.(PMID:30371208) Reduced baroreflex control is related to poor patient outcomes after stroke, independent of absolute blood pressure.(PMID:19834010) Disrupted autonomic control also promotes infection, an important determinant of outcome.(PMID:26723020)

Reversing baroreflex and vagal dysfunction is, therefore, widely held to have the potential to improve cardiovascular control and patient outcome in this context.(PMID:19834010) Non-invasive peripheral neuromodulation restores autonomic control. Vagal nerve stimulation improves autonomic control and reverses baroreflex dysfunction (PMIDs:28949064) but this has previously required surgically implanted devices which are expensive and impractical in the context of acute stroke. Afferent Electronic have achieved the same effect as these implantable devices by non-invasive transcutaneous autonomic neuromodulation (TAN). We have used this simple, safe, hand-held, low-cost device to increase vagal activity and baroreflex sensitivity through non-invasive, painless stimulation of nerves located in the outer ear to control blood pressure.

We have demonstrated that baroreflex sensitivity can be increased at the bedside by TAN for 30 minutes following acute trauma (a patient group in whom baroreflex dysfunction also develops rapidly after injury) If this can be replicated in thrombectomy patients, it will aid recovery and rehabilitation through five complementary mechanisms where it has been clinically demonstrated that increasing vagal nerve activity:

- Restore baroreflex sensitivity;
- Increase blood flow to ischaemic brain tissue through vagal activation.(PMID:27357059);
- Dampen cerebral/systemic inflammation.(PMID:26723020);
- Reduce atrial fibrillation and myocardial injury,(PMIDs:5744003,22739118) which are common after stroke, and independently predict cognitive decline and cardiovascular mortality
- Allows immediate commencement of vagal nerve stimulation, which has recently been shown to enhance upper-limb rehabilitation.(PMID:33894832)

Efficacy/ Safety/ Tolerability

In a systematic review, no safety or tolerability issues were identified. In our recent study, 3/86 participants reported minor outer ear irritation; no major adverse events noted.

1.2 Rationale for study design

Afferent Electronic's proof-of-concept data shows daily TAN reduces BP and BP variability lasting several months even in drug-resistant hypertensive patients (https://fundingawards.nihr.ac.uk/award/NIHR202116). In this proof-of-concept randomised sham-controlled trial, this study will examine whether early TAN on presentation for mechanical thrombectomy improves autonomic function in patients with acute ischaemic stroke by reducing blood pressure lability. Better control of blood pressure early after ischaemic stroke is challenging with drug therapy, due to compliance and ability to ingest tablets. Our investigational device might be an improvement on currently available pharmacological treatments, since it is non-pharmacological and is readily accessible to all patients including after discharge from hospital. Compliance with device use can be monitored remotely to optimize therapeutic effect. We will examine the effect of TAN versus sham stimulation to ensure any effects are not attributable to placebo effect. The intervention has no impact on standard of care with other (pharmacological) therapies.

1.3 Assessment and management of risk

The only established risk of the procedure is local skin irritation to the outer ear region. There is a theoretical risk of autonomic-induced cardiac arrhythmias, but there are no reports from an extensive literature search. The battery operated device bears no electrical risk.(PMID: 34801224) There are no other neurological side effects that have been reported. Our recent experience in 86 patients admitted with orthopaedic trauma who underwent TAN for pain control demonstrate no risks other than a local sensation of tingling in the outer ear and mild, inconsequential skin irritation that did not require any clinical intervention/therapy. The device is located well away from implanted pacemakers so pose no risk to other indwelling electrical devices. The overall risk-benefit ratio is very low, less than pharmacological interventions in stroke for blood pressure control.

2.0 Trial objectives

2.1 Primary objective(s)

To determine whether non-invasive transcutaneous autonomic neuromodulation results in lower blood pressure variability during the first 24 hours from the start of mechanical thrombectomy, as measured by coefficient of variation compared to the control treatment.

2.2 Secondary objective(s)

- Systolic blood pressure variability in the first 24 hours from the start of mechanical thrombectomy
- Diastolic blood pressure variability in the first 24 hours from the start of mechanical thrombectomy
- Heart rate variability in the first 24 hours from the start of mechanical thrombectomy
- Neurological recovery NIH Stroke
- Arrhythmias
- Myocardial injury

2.2.1. Future research

- Biomarkers for risk of infection

2.3 Endpoints

2.3.1 Primary endpoint(s)

Coefficient of variation of systolic blood pressure measured in each patient during the first 24 hours from the start of mechanical thrombectomy.

2.3.2 Secondary endpoint(s)

- Systolic blood pressure variability from blood pressure measured in each patient during the first 24 hours from the start of mechanical thrombectomy.
- Disatolic blood pressure variability from blood pressure measured in each patient during the first 24 hours from the start of mechanical thrombectomy.
- Heart rate variability measured during each period of transcutaneous autonomic neuromodulation.
- NIH Stroke Scale (NIHSS) measured in each patient before the start of mechanical thrombectomy and 24 hours after the start of procedure.
- Clinically recorded arrhythmias in each patient during the first 24 hours after the start of mechanical thrombectomy.
- Absolute levels of serum high-sensitivity troponin-T levels measured before the start of the first transcutaneous autonomic neuromodulation and at the end of the second transcutaneous autonomic neuromodulation.

** please see Appendix A for details on the assessment of primary and secondary outcomes*

2.5 Study design

This is a phase IIa, Triple-blind, sham-controlled randomised trial of superiority (stimulation is superior to sham treatment).

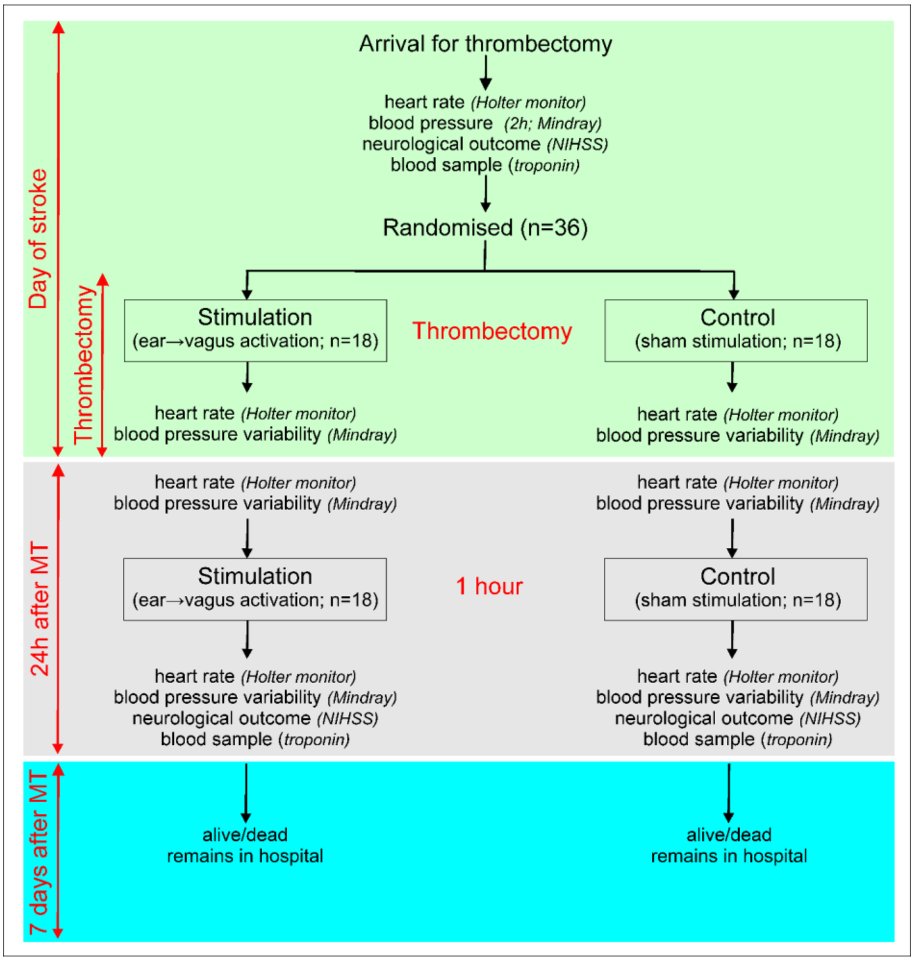

2.6 Study setting

This is a single centre UK study taking place within Barts Health NHS Trust. Provision of mechanical thrombectomy is the only site-specific requirement to run the study. Potential participants will be identified through two possible routes: 1) when referrals for mechanical thrombectomy are made by the stroke consultants or 2) from outside referring secondary care, NHS hospital centers.

3.0 Patient Evaluability and Replacement

Withdrawn participants will not be replaced (intention to treat analysis).

3.1 Target Accrual

36 participants undergoing mechanical thrombectomy will be recruited.

3.2 Participant identification and recruitment

The stroke consultant (direct care team) receiving referrals for mechanical thrombectomy will screen potential participants for eligibility at the hospital site. Once eligibility is established, the stroke medicine consultant will confirm with the interventional radiology consultant the participant is eligible to enter the trial. The eligibility confirmation will be documented in the medical records prior to randomisation. Participants that undergo screening and meet the inclusion criteria will be recorded on the study screening log stored in the investigator site file. Only anonymised screening data will be collected by the central trial coordinating team for publication purposes. After provision of written consent, the patient has been enrolled in the study and recorded on the study enrolment log with their trial ID.

4.0 Informed consent procedures

Patients presenting with acute ischaemic stroke are frequently unable to consent for themselves due to neurological impairment, sedation or delirium. For every 30-minute delay, the odds of achieving a good outcome are reduced by 50% from initial confirmation of stroke to thrombectomy achieving reperfusion. (PMID: 26956258) Therefore, due to the emergency clinical situation, VANS will adopt a research without prior consent (also referred to as deferred consent) where eligible patients will be enrolled in the study through consent from a Professional consultee who would be the named interventional radiology consultant in charge of the patient's thrombectomy care. The consultant will be asked to sign the Consultee Declaration Form. The consultant retains one copy of the form; one copy goes in the participant's medical notes and the third in the investigator site file.

The Principal Investigator retains overall responsibility for the informed consent of participants at their site and must ensure that any person delegated responsibility to participate in the informed consent process is authorised, trained and competent to participate according to the protocol and standards set by GCP. Delegation can only be to medically qualified research team members.

Following participant enrolment, a delegated member of the site research team will approach the patient's Personal consultee if available. This may be a family member, partner or friend. This Personal consultee will be fully informed about the trial by a member of the research team or the responsible clinician. Personal Consultees will be given a copy of the Personal Consultee Information Sheet. They will be asked to provide their opinion as to whether the patient would object in taking part in medical research. If they decide that the patient would not object they will be asked to sign the appropriate informed consent form. Two copies of this form will be made – one copy in the medical records, original in the investigator site file and one left with the representative.

If a Personal Consultee advises that, in their opinion, the patient would not choose to participate in the trial, then the trial treatment will be stopped (if ongoing). The Personal Consultee will be asked whether, in their opinion, the patient would be willing to continue with ongoing data collection. If a Personal consultee is not available during the trial period, the data collected will be retained and the seven day follow-up data will be collected from reviewing the patients medical records at Barts NHS Trust.

In cases where consent has been given by a Personal or Professional consultee, the patient will be informed of their participation in the study as soon as they have return of capacity. If the patient has regained capacity during their stay in Barts NHS Trust, a member of the research team will meet with them and explain the study and provide a copy of the patient information sheet. The patient will need to sign a copy of the informed consent form. One signed copy will be retained by the patient, one placed in the medical notes and one in the study site folder. If participants decline to participate in the trial, consent will be sought to retain and analyse their data and samples collected so far.

If the patient does not regain capacity during their stay at Barts NHS Trust and transferred, to another hospital without providing consent a covering letter notifying them of their participation in the trial, participant information sheet and consent form will be sent with their medical notes.

On the occasions patients have regained capacity and are discharged before being approached by the research team they will be contacted in the first instance by phone. If the research team do not get a response to at least three telephone call attempts, then the patient will be approached by post a maximum of twice. The patient will be sent a covering letter and a copy of the patient information sheet and consent form. The letter will direct the patient to the participant information sheet for detailed information on the trial and provide contact details for if the patient wishes to discuss the trial further.

The right of a participant to refuse participation without giving reasons will be respected. The participant will remain free to withdraw at any time from the study without giving reasons and without prejudicing their further treatment and will be provided with a contact point where they may obtain further information about the study. Where a participant is required to re-consent (for example if new Research Safety Information becomes available during the study, or following an amendment that affects the participant, or new information needs to be provided to a participant) it is the responsibility of the PI to ensure this is done in a timely manner and prior to the next use of the investigational device (where applicable).

Patients who are consented but not entered into this study should be recorded (including reason not entered) on the screening log in the Investigator Site File (ISF). Original signed consent forms will be kept by the investigators and a copy will be given to the participant or representative.

4.2 Vulnerable participant considerations

The study will involve the participation of vulnerable participants, as adults with acute ischaemic stroke are frequently not able to consent for themselves. The PI is responsible for ensuring that all vulnerable participants are protected and participate voluntarily in an environment free from coercion or undue influence.

4.3 Writing, reading, and translation considerations
If verbal translation is needed, an NHS hospital interpreter will be provided.

4.4 Participants lacking capacity
Participants presenting with acute ischaemic stroke frequently lack the capacity to consent for themselves. Please see above section for description of process. If a participant is able to consent for a Clinical Investigation but later becomes incapacitated, the original consent given endures the loss of capacity, because the study has not significantly altered.

4.5 Consent for ancilliary studies
Informed consent will be obtained from participants for their blood samples to be used for any future research associated with the trial.

5.0 Participant eligibility criteria

5.1 Inclusion criteria
- Age 18 years and over
- Requiring mechanical thrombectomy for acute ischaemic stroke under general anaesthesia and/or sedation
- Established hypertensive and/or hypertensive on admission for mechanical thrombectomy (systolic BP >140mmHg; diastolic BP >80mmHg)

5.2 Exclusion criteria
- Refusal to provide informed consent
- Current participation in a clinical trial of a treatment with a similar biological mechanism.
- Previous enrolment into VANS trial.
- Anatomical or other contraindication to non-invasive transcutaneous autonomic neuromodulation (see Investigator Brochure appendix D).
- Pregnancy.

6.0 Study Schedule

6.1 Schedule of treatment for each visit
Visit 1 (eligibility check and consent before mechanical thrombectomy)
- On referral for mechanical thrombectomy, potential participants will be screened for eligibility and if suitable informed consent will be obtained. Please see section 4 for further details on informed consent procedures.
- Routine pre-procedure serum pregnancy test for female participants will be checked.
- Demographic information, medical history, current concomitant medication, NIHSS will be recorded.

Visit 2 (day of mechanical thrombectomy)

- Following informed consent, participants will be randomised to either the active stimulation or sham arm and assigned a trial ID.
- Heart rate data will be captured by dedicated Holter monitors.
- Blood samples will be collected before the start of mechanical thrombectomy.
- Before the sterile mechanical thrombectomy field is prepared, the AffeX-CT device will be applied to each ear for the duration of the mechanical thrombectomy procedure. Leads will be placed on each ear in the tragus area of the outer ear. The device will be preset to deliver either sham or active TAN. The ear leads will remain attached for the duration of the thrombectomy procedure which is typically two hours and removed at the point the patient leaves the radiology suite to be taken to the post recovery anaesthetic room.

Visit 3 (day after mechanical thrombectomy)

- The AffeX-CT device will be applied to each ear for typically one hour during this period
- Heart rate data will be captured by dedicated Holter monitors.
- Study data will be collected including concomitant medication and NIHSS will be recorded.
- Blood samples blood will be collected after the one hour stimulation period.
- Blood pressure data will be downloaded from the electronic health record and stored securely within QMUL/Barts Health NHS Trust for subsequent analysis.

Visit 4 (seven days after mechanical thrombectomy)

- Patient vital status will be checked (alive/ dead/ in-hospital)

6.2 Schedule of assessment

Event/Visit	Screening	Day of MT*	Morning after MT	24 hours after MT*	Day 7 after MT*
Inclusion/exclusion	x				
Heart rate and blood pressure safety review		x		x	x
Informed Consent (professional/ personal)		x		x	
Informed Consent (patient)					x
Randomisation		x			
Heart rate		x	x		
Blood pressure		x		x	
Investigational procedure: TAN		x	x		
Medical record review	x	x		x	x
Trial blood sample		x		x	
Follow-up					x
Safety review		x		x	x

**MT: Mechanical thrombectomy*

6.3 Randomisation procedure

Randomisation will occur on the day of mechanical thrombectomy after informed consent but before the procedure is due to start. Participants will be centrally allocated to either sham or active stimulation in a 1:1 ratio with block randomisation. No minimisation is required. The allocation sequence is generated by the Statistician which will then be provided to the device manufacturer (Afferent) who will be programming the simulators accordingly. The randomisation information will be concealed to all trial investigators. The stimulators will be numbered sequentially starting at 1, and programmed according to the random allocation sequence provided. There will not be a possibility to identify the treatment group by any markings on the stimulator boxes as the patients will be enrolled on the database.

To register a patient into the VANS trial, research staff at the site will log on to a secure data entry platform hosted by Queen Mary University of London and complete the patient's details to obtain a unique patient identification number. The patients will be allocated to a sequential trial group ID corresponding to the stimulator box, starting with 1, then 2 and so on. The system for generating the allocation sequence will be bespoke and will be developed in–house. The system will be validated for use by the trial statistician. All details of the eligibility screening process including participants who are screened and not randomised will be documented in the medical records.

6.4 Blinding

This is a triple-blind trial, with trial participants, care providers and endpoint assessors to the treatment group allocation. The primary outcome will be analysed by individuals masked to the patient treatment allocation and clinical outcomes. Afferent Medical Solutions Ltd will be unblinded to facilitate reporting of Serious Adverse Device Events (SADE) or Unexpected Serious Adverse Device Events (USADEs) to the MHRA. The blinded interventions are highly comparable, as the same device will be used for all participants randomized to either active stimulation or turned off. Final unblinding of all study participants will occur after the data set is locked.

6.5 Unblinding

Unblinding a participant's treatment is strongly discouraged as it can compromise the integrity of the trial. However, unblinding may be necessary on rare occasions for medical or safety reasons that can affect clinical management. In this trial, unblinding may be required if a patient experiences a cardiac arrhythmia of concern to attending clinicians. The PI or assigned delegate must determine the necessity of unblinding, and site teams can contact the study coordinating team for further discussion. Healthcare professionals will be provided with emergency contact details of the research team to quickly unblind a participant if knowledge of the treatment assignment is essential for optimal medical treatment. A 24-hour emergency unblinding procedure will be available, and participants will receive a trial card with emergency contact details. Unblinding requests are expected to be rare as the rescue treatment should not depend on the treatment group allocation.

To ensure the safe unblinding of information in case of a safety concern, we will store unblinding details in a securely locked cupboard that can be accessed by the research/clinical team with prior discussion and approval from the PI or their designated representative. The cupboard will be accessible 24/7. Unblinding information will be contained in opaque sealed envelopes that will be

labelled with the corresponding stimulator box number and treatment group allocation (stimulation or sham). The Statistician will be responsible for inserting the treatment group information and sealing each envelope, which will be witnessed and verified by an independent staff member not involved in the trial. This process will ensure that the treatment group allocation cannot be viewed without opening the sealed envelope.
To enhance the security and confidentiality of the unblinding process, each envelope will be signed by the Statistician on the back, and they can be examined at the end of the trial to verify that none of them have been tampered with. The envelopes will be kept in a designated folder that is labelled clearly, and this folder will be stored in a securely locked cupboard that can be accessed at any time of the day. Additionally, both the Statistician and the witness will sign a file note that outlines the process used to create and seal the envelopes.
Treatment identification information should be kept confidential and should be disseminated only to those individuals involved with the medical management of the participant. The Chief Investigator and Principal Investigator will be kept informed of all instances of unblinding but should remain blinded to treatment allocations themselves. The Trial Manager and the site staff will maintain a record of all unblinding events including: patient trial ID, the date code break was performed, the person who broke the blind, and reason for it. The breaking of the code and the reasons for doing so will also be captured on the electronic case report form (eCRF), in the site file and medical notes. The code break for the trial will be held by the trial coordinator until the end of the trial when it will be filed in the Trial Master File (TMF). The Chief Investigator will ensure any unblinding is documented at the end of the study in any final study report and/or statistical report. The information will also be disseminated to the Trial steering Committee (TSC).

6.6 Study assessments

The following data will be collected from all patients:

Randomisation data

- Initials
- Date of consent and mechanical thrombectomy
- Age
- Sex
- Ethnicity
- Trial ID (generated automatically at the point of randomisation)

Baseline data

- Ethnicity
- Medical history
- Smoking status (within the last 14 days)
- Cardiovascular medications
- Laboratory values (haemoglobin, creatinine, neutrophil/lymphocyte count, albumin)
- NIHSS on admission to Royal London Hospital
- Systolic and diastolic blood pressure
- Blood sample
- Pre mechanical thrombectomy resting heart rate
- Planned level of care on the first night after mechanical thrombectomy
- Safety review

During mechanical thrombectomy

- Start and end times of mechanical thrombectomy
- Start and end times of TAN
- Anaesthetic technique (general anaesthesia/sedation)
- Clinically recorded arrhythmias
- Heart rate (Holter) during first intervention period
- Systolic blood pressure
- Diastolic blood pressure
- Level of care
- Safety review

24 hours after the start of mechanical thrombectomy

- NIHSS
- Start and end times of TAN
- Clinically recorded arrhythmias
- Blood sample after the end of TAN stimulation period
- Cardiovascular medications
- Level of care
- Laboratory values, if available (haemoglobin, creatinine, neutrophil count, lymphocyte count, albumin)
- Heart rate (Holter) during second intervention period
- Systolic blood pressure
- Diastolic blood pressure
- Hospital discharge (if applicable)
- Safety review

Day seven follow-up

- Mortality status and in hospital stay seven days after mechanical thrombectomy
- Safety review

6.7 Trial intervention

Each participant will have the Affex-CT device connected to both ears via electrical leads. The device will be preset to deliver either transcutaneous autonomic neuromodulation or no stimulation. In the treatment arm, the device will be turned 'On' by a trained member of the research team and the transcutaneous autonomic neuromodulation will be applied continuously until the end of the stimulation period when the device will be turned 'Off'. For the control arm, no stimulation will be applied.

The intervention will be administered across two time points in each participant. The first will be delivered for the entire duration of the mechanical thrombectomy procedure. The start of mechanical thrombectomy is defined at induction of anaesthesia which is the patient's arrival in the anaesthetic room but before being wheeled into the radiology suite. The end of mechanical thrombectomy is defined as the point the patient leaves the radiology suite to be taken to the post recovery anaesthetic

room. The second will be delivered on the morning after mechanical thrombectomy regardless of the time when the first procedure was administered. This will happen typically in the morning between 0800-1200h for one hour (± 10 minutes). The duration of participation is seven days from the start of the mechanical thrombectomy procedure for each participant. No special dietary or 'life-style' requirements will be imposed (this study will gather efficacy data for the intervention while establishing the feasibility of a full-scale trial).

6.8 Follow-up procedures

Follow-up is completed seven days after mechanical thrombectomy. The minimum follow-up period following the last use of the investigational device will be six days after the last use of the device, given the performance characteristics, safety and effectiveness of the investigational device. Follow-up will be completed by either reviewing the patient's medical records or calling the patient.

6.9 Compliance

Withdrawal and protocol deviations will be recorded. Non-compliance or missed interventions will be confirmed by the device which records periods of stimulation/activation.

7.0 Participant, study, and site discontinuation

It is always within the remit of the responsible clinician to withdraw the participant from the study for appropriate medical reasons. This can be (but is not limited to) individual adverse events, new information gained about a treatment, or if it is felt to be in the participant's best interest. Specific circumstances where participants will be withdrawn from the study are if the auricular stimulation site became inflamed and/or painful.

- If a participant's Personal or Professional consultee wishes to withdraw, data collection and follow-up would continue for withdrawn participants enabling these data to still be used.
- No further blood samples would be collected for withdrawn participants.
- The reasons for participant withdrawal and follow-up information collected would be recorded and timed.
- Participants will not be replaced.
- Withdrawn participants could not be re-entered on this study.
- Clinical data for participants or their representatives that have withdrawn from the study will be kept only up until the date they withdrew, unless they also request for their study data to be deleted.
- Already collected samples and data would be used [intention-to treat analysis].
- Participants who have become lost to follow-up by seven days will be documented.

8.0 Laboratories and samples

Results from standard of care clinical Barts Health laboratory tests will be recorded. The analysis of troponin measurements will be made from frozen blood samples by The Doctors Laboratory using the Roche high-sensitivity troponin assay
(see: https://labogids.sintmaria.be/sites/default/files/files/troponin_t_hs_2019-02_v8.pdf) and future research studies.

8.1 Central laboratories

The Doctors Laboratory (vendor, London WC1) for high-sensitivity troponin measurement.

8.2 Local laboratories

Results from standard of care clinical Barts Health laboratory tests will be recorded.

8.3 Sample collection, labelling, and logging

All blood samples will be pseudo-anonymised. Samples collected at each participating site will be labelled with the participant's corresponding trial ID and kept in a hospital freezer at an optimal temperature for the troponin assay until collection. The samples will be routinely collected and transferred to WHRI where they will be stored prior to transfer to The Doctor's laboratory. The full sample, collection, labelling, logging and transfer procedure will be documented in the study laboratory log. The trial coordinating team will provide sites with a SOP on sample collection, processing and storage.

8.4 Sample transfer, chain of custody, and accountability
Handling of the samples upon arrival at the local and central laboratory will be documented. All samples will be logged upon receipt and the laboratory will ensure that the physical integrity of these samples have not been compromised in transit. If compromise has occurred, the trial coordinating team, as well as the Sponsor, will be informed of this. Upon receipt of samples, laboratory staff will ensure that all samples are accounted for as per the labelling.

8.5 Sample Storage Procedures
The samples will be stored in pseudo-anonymised form with the participant's trial ID written on the label in a local hospital freezer at an optimal temperature for the troponin assay until collection. The samples should be put in the freezer within two hours of preparation. The samples will not be destroyed if a patient withdraws from the study unless they specifically request so. If the patient requests for the samples to be destroyed the Tissue Custodian (Chief Investigator), will inform the lab who will ensure the samples are destructed as per the Human Tissue Act. This will be documented in the TMF and ISF of the participating site.

8.6 Sample and result recording and reporting
Troponin-T will be measured by the Doctors Laboratory and shared with the Chief Investigator by secure electronic communication after the last patient sample has been analysed.

8.7 Sample management at end of study
The samples will be stored beyond the end of the trial to be used for closely related studies in the future. After completion of any potential sub-studies the samples will be destroyed according to the Human Tissue Authority's Code of Practice.

9.0 Investigational Device

9.1 Name and description of Investigational Device
A full description of the investigational device AffeX-CT to be used is provided in Investigator Brochure version 1.

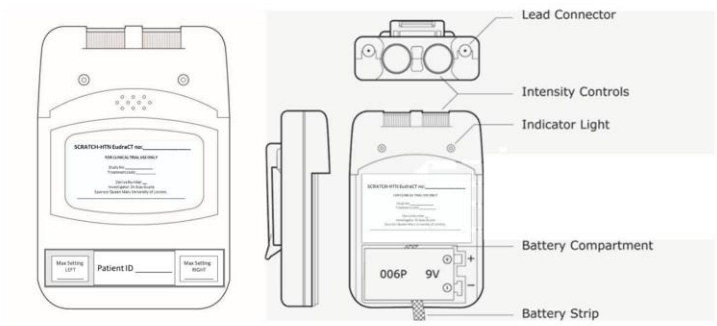

The device is manufactured using standard electronic components, pbc assembly processes and ABS (Acrylonitrile butadiene styrene) for the casing, plus ear-clips comprised of moulded plastic parts with conductive rubber electrodes. Ear-clips are the only parts of the device that will be in contact with human aurciular tissue. *The AffeX* device is a hand-held, battery (9V) operated, portable device consisting of a signal-generating, amplifying electronics, control dials for patients to adjust the stimulation intensity. A pair of stimulation electrodes in the form of ear-clips are used to apply the electrical signal to the skin (stimulation surface) of the tragi bilaterally. *AffeX* device produces a fixed electric current signal consisting of 30 Hz biphasic asymmetrical pulses with 200 µs duration of the positive phase. The current waveform has an adjustable amplitude of between 0 and 80 mA. Usually, the stimulation is applied with the intensity between 1-4 mA. The signal is transmitted through the skin of the tragus to the subcutaneous nerves. Each stimulation is designed to be applied for at least 60 min daily.

9.2 Intended Performance

Investigation Brochure appendix B details Instructions for Use of the investigational device. AffeX-CT has been set to deliver a biphasic pulse with the following parameters: The performance of each device has been validated to meet the Intended Performance specification.

Waveform parameters:

- Channel: Dual, isolated between channels
- Mode of operation: continuous,
- Pulse intensity: recommended range for the investigation is 0.1-8mA (device range is 0-80mA)
- Pulse Rate: 30 Hz
- Pulse width: 200 µsec
- Duration /Time: Continuous for 30min
- Wave Form: Bi-Phasic Asymmetrical square pulse
- Electrical specification:
- Battery 9v (6F22)
- Mechanical specification:
- Dimensions: 95(H) x 60(W) x 23(T) mm
- Weight: 115 grams (inc. battery)

Formatted: French

10.0 Legal status of Investigational Device

AffeX-CT device has not been approved by the MHRA for use as "a non-implanted, non-invasive transcutaneous autonomic neuromodulation device for the functional management of the arterial blood pressure" (Intended Use).

It has been supplied for ‘research only’ use as "a non-implanted, non-invasive transcutaneous autonomic neuromodulation device for the functional management of the arterial blood pressure" (Intended Use). The AffeX-CT device is a TENS device (Totally TENS, model WL-2103A/TT-21AL) on which it is based has been marketed as an Over-The-Counter (OTC) TENS device (Intended Use: (with

a clinical prescription) for the symptomatic relief and management of chronic (long term) pain. No adverse events have been reported.

The trial device falls within the European Medical Device Regulations (MDR) as a Class IIa device in line with other TENS devices and Class II device by the FDA. The AffeX-CT device uses the Totally TENS, model WL-2103A/TT-21AL product which has been designed and manufactured to ISO13485 Quality Management System. The device is manufactured using standard electronic components, pcb assembly processes and ABS (Acrylonitrile butadiene styrene) for the casing. Ear-clips use moulded plastic parts with conductive silicone rubber electrodes. Each device is designed for use by one participant only and must not be re-used by other participants. There will be in total 36 individual investigational devices supplied for the trial, one for each participant and 12 devices as replacements devices should devices issued to participant fail or malfunction. Each device is marked with a unique identification number. The electrodes supplied with the device will also be marked with the device's unique identification number. Additional devices will be supplied for training purposes and in reserve, if any participants encounter issues or cases of malfunction.

The device is compliant with the following electrical, mechanical and safety standards:
Mechanical:

- Robustness EN 60601-1 (Classification IP22)
- Water resistance EN60529 (Classification IP22)
- Ingres protection EN60529 (Classification IP22)

Electrical:

- EMC susceptibility / immunity IEC 60601-1-2/60601-1-2
- Low voltage safety IEC 60601-1-2/60601-1-2
- Quality Management System used: ISO 13485: 2016

10.1 Device Manufacturer(s) and supply arrangements

For the purpose of the VANS study, the AffeX-CT device is being used 'off-label' and Afferent Medical Solutions Ltd will assume responsibilities of 'the manufacturer' and must therefore fulfil all the requirements of a manufacturer as set out in the UK Medical Devices Regulations 2002, including notification of a clinical investigation to the MHRA.

Named supplier (Afferent Medical Solutions) (''Afferent'') will assume responsibility for:

- manufacturing
- labelling in accordance with the medical device's regulations 2002, ISO14155 and GCP
- separately testing and calibrating each device prior to the delivery to the trial team
- delivering the investigational units to the research facility
- assuming management of and providing maintenance services
- each device will have a unique identification number which will appear on the device labels. Their associated ear-clip & leads and carry case will also have an identification label with the same identification number as the device

All devise deficacy and event reporting to the MHRA

- disposal of devices

The Sponsor will be responsible for the management of collecting all units upon trial completion and their return to Afferent Medical Solutions (see CIP section 10.9 destruction, return, and recall devices). An approved courier service will transport the investigational devices to Royal London Hospital. A log recording receipt of the medical devices will be kept. Devices will be returned to the manufacturer at the end of the trial by courier.

Supplier information

**Company**	**Afferent Medical Solution Ltd**		
**Register Office**	4 Willoughby Drive, Solihull, England, B91 3GB	**Company No**	**12126455**
**Contact Person**	Dr Everard Mascarenhas	**Tel**	07815 444305
**Address**	6 Almond Av, Ickenham, England UB10 8NA	**email**	ev.mascarenhas@afferentmedicalsolutions.com
**Technical Consultant/scientific advisor**	Prof Alexander Gourine	**email**	a.g@afferentmedicalsolutions.com
**Regulatory Consultant**	Dr George Zajicek	**email**	george.zajicek@hotmail.co.uk
**Medical Advisor**	Dr Andrey Gourine	**email**	a.gourine@afferentmedicalsolutions.com

10.2 Investigational Device Accessories

AffeX-CT is a standalone device and supplied as following:

- Control Unit
- 4x Ear-clips and connecting leads
- Battery (6F22)
- User Guide
- Instructions for Use (IFU) (see Investigator Brochure appendix B)
- Storage case (soft bag)

10.3 Packaging and labelling of Investigational Devices

The devices will be packaged and labelled in accordance with the Medical Devices Regulations 2002 and ISO14155 GCP. No special requirements for this non-sterile product are necessary.

Each device will have the following labels on the front cover:

Front cover of device

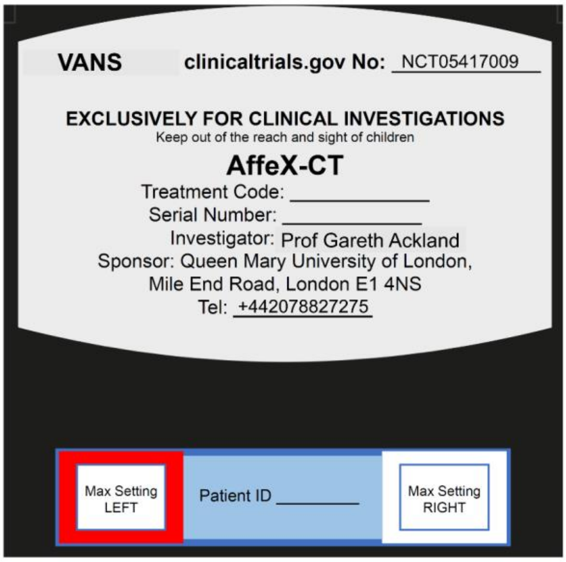

Back cover of device

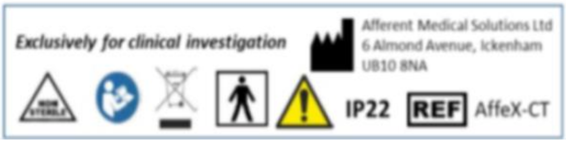

Refer to Investigator Brochure appendix A for more information on Symbols and Nomenclature Description. The carry case will have the information appearing on the front cover label printed on it. AffeX-CT is a non-sterile product.

There are no special requirements for packaging. Device will be labelled in accordance with medical device regulations 2002 and ISO 14155 GCP (see Investigator Brochure 10.5; appendix E). Front labels are to be colour coded; range of colours for participants and white labelled for demonstration/training devices.

The ear-clips will be colour coded, white and black and red and black to allow users to identify which ear-clip should be attached to the left and right ear.

10.4 Accountability and Traceability

Each investigational device will be assigned a unique identification number. The Chief Investigator and investigator sites will maintain accountability logs at sites and delivery records to record the dates when:

- devices were received, the name of the person who received them and the quantity received
- each device was used, who used it and the trial identifier of the participant it was used on.

- devices were returned including the name of the person who returned or disposed of the device.
- any devices were returned because they were unused, expired or malfunctioning.

Lot numbers, batch numbers and/or serial numbers will be used to trace devices.

10.5 Assessment of compliance

Each investigational device monitors and records delivery of programmed current, thus storing usage information internally. Treatment non-compliance will be documented by the investigator and reported to the Sponsor. There is no minimum compliance threshold. All non-compliant participants are followed up.

10.6 Device storage

Each investigational devices will be stored in a locked cupboard within the Critical Care and Perioperative Medicine research group secure storage site. Devices held at the research site must be stored in a secure location (e.g., locked cupboard), with access limited strictly to members of the investigation team.

Only members of the investigation teams will have access to the investigational devices. No special storage requirements are necessary for the device.

After use, participants will be urged to unplug the ear clip leads from the unit, and place all components back into the sachet/casing, in such a way, as to minimise the chance of accidental removal or damage (e.g., drawer or shelf)

10.7 Device training and experience requirements

Prior to the trial start, a training session will be conducted by an Afferent trainer for members of the Investigation site team. In case of any additional inquiries, contact details to a delegated Afferent representative, as well as the trainer, will be provided, to respond to any requests and for provision of any additional information. Investigation site team will be responsible for training participants in the use of the device. They will also be required to follow a set training routine, which will include an assessment of the participant's ability to satisfactorily use the device. This will be documented on a training log.

10.8 Administration of investigational device

Only trained research staff will operate the device. TAN treatment is started by attaching an electrode clip to the tragus of each ear and switching the stimulation 'ON' by turning the dial from the off position (marked as 0 on the dial) to position 4 or 5 on the dial. Once the patient experiences a tingling sensation, the signal amplitude is reduced by turning the dial anti-clockwise for 1 division below the tingling sensation threshold. The device will then continue to apply the stimulation for a fixed duration. The electrode must remain attached to the tragus of the ear for the entire treatment session. At the end of the stimulation period, the device will automatically stop the treatment session. The

jack-plug of each electrode is inserted into the socket of the device control unit and clipped onto the tragus. It is important that both electrodes are connected, one for each ear.

10.9 Destruction, return, and recall devices

All units are to be returned to Afferent Medical Solutions upon trial completion. Afferent will arrange for the disposal of all the units in an appropriate and controlled manner, upon the request from the Chief Investigator following accountability checks by the allocated monitor. The Sponsor should return the units to Afferent Medical Solutions Ltd, 6 Almond Avenue, Ickenham UB10 8NA, UK. Separate ear-clips will be used to train participants and are to be disposed of by the Research Nurse in accordance with the clinical unit's standard operating procedures.

Regulations require that disposal of electrical equipment, including used and unused medical devices, is handled in a controlled manner. Devices that may be contaminated after use or that may contain chemicals or elements that may be hazardous to people or the environment will be disposed of in accordance with the applicable government regulations. Afferent will be responsible for the disposal of all devices and will ensure their disposal complies with Restrictions of Hazardous Substances (RoHS) and in accordance with the applicable government regulations. In case of a device recall, Afferent will be responsible for coordinating with the investigator and managing the process in a controlled manner.

10.10 Usage schedules

- First administration will be for duration of mechanical thrombectomy [which typically lasts two hours]
- Second administration will be the morning after the mechanical thrombectomy [0800-1200h] which typically lasts one hour.
- No changes to usage schedule are required for different age groups or demographics.

10.11 Usage modifications and delays

- It is not envisaged that any modification in device usage or device itself during the conduct of the trial, except if a person develops local irritation/dermatitis which makes it difficult to use the clips to the ear. In that case, we will withhold treatment until the person can use the device or try to consider alternative options.
- There are no adverse events that could require the device usage to be modified.
- If participants develop cardiac arrhythmias, the device will not be used during that treatment period.
- Device usage will not be modified in accordance with the participants' results.
- The device setting/dose is modified to minimise local ear discomfort [i.e., current reduced on participant request].
- If the first treatment period is interrupted for clinical or device-related reasons, the Principal Investigator will be informed; proceeding with further treatment on the following day will be permitted as long as there are no [new] exclusion criteria met and after consultation with the Chief Investigator.
- The hardware of the device will not need to be modified during the study duration.

The most likely scenario we anticipate is the interruption of a treatment session. It is possible that patient stops the device or removes the electrode clips from the ear(s) before the duration of the session is complete. In that case, we will advise to reattach the device again for the remaining period and record the interruption. If they are not able to use the device for the full stimulation period, we will record a protocol deviation.

10.12 Management of device-specific adverse events

Any device-specific adverse events (AEs) will be recorded in the patient medical records and eCRF and the information – in pseudo anonymised format – will be passed on to the delegated representative from Afferent Medical Solutions Ltd.

All AEs will be informed to the device manufacturer (Afferent Medical Solution Ltd) as soon as possible after the investigation site staff has become aware of the event. Details of the device-specific adverse events will be sent to the Afferent team on the following email a.gourine@afferentmedicalsolutions.com, and where possible, a member of the trial team will contact the Afferent team via phone at 07724578883.

We anticipate following side effects associated with TAN applied via electrical stimulation for the tragus:

- Light-headedness
- Fatigue/tiredness
- Mood changes
- Neck pain
- Tooth pain
- Pain/local skin irritation due to attachment of the device ear clips
- Tingling sensation due to the use of the device
- Palpitations

AEs will be recorded in the medical notes and on the appropriate AE pages of the CRF corresponding to the visit or interaction with the study team. Serious adverse events (SAEs) require expedited reporting to the device manufacturer (Afferent Medical Solution Ltd) and the Sponsor or designee regardless of relationship to study device or study procedure (refer for more information to Investigators Brochure 9.3). All those will be informed as soon as possible and within 24 hours of the Investigator/ Investigation site staff become aware of the event. Potential interactions with other therapies any Device related SAEs are not anticipated. However, if they occur, the Principal Investigator and Investigation site will follow Afferents SOPs for reporting and managing them. Please see CIP section 13 for further information.

10.13 Potential interactions with other therapies

We do not anticipate any adverse interactions between existing medications and TAN treatment, except it is possible that those on beta-blockers may have an increased blood pressure lowering effect with the use of device. This is because TAN is likely to augment vagal tone, whereas beta-blockers inhibit sympathetic effects, and both of these could be additive for the patient. Similarly, drugs that

increase the parasympathetic stimulation, such as clonidine may also increase the blood pressure efficacy of the device. However, clonidine is rarely used nowadays, and certainly not amongst first line medications for the treatment of high blood pressure.

10.14 Management of incorrect usage

The product has been designed for use by only one patient. It must not be re-used by other patients. If the product fails to operate as described by the IFU (Appendix B) or malfunctions in any way a Product Complaint Reports as set out in Appendix G should be filed and submitted directly to Afferent Medical Solutions Ltd (ev.mascarenhas@afferentmedicalsolutions.com).

10.15 Precautions regarding contraception

Pregnancy is an exclusion criteria.

10.16 Arrangements for post-study access to investigational device

We will follow-up all participants for a period of seven days after the completion of the last stimulation period, no further access is possible

11.0 Safety Reporting

11.1 Site investigator assessment

The Principal Investigator is responsible for the care of the participant, or in their absence an authorised medic within the research team is responsible for assessment of any event for:

- Seriousness
  Assessing whether the event is serious according to the definitions given in section **Error! Reference source not found.**.
- Causality
  Assessing the causality of all serious adverse events in relation to the study treatment according to the definition given. If the SAE is assessed as having a reasonable causal relationship with the investigational device, then it is defined as an ADE.
- Expectedness
  Assessing the expectedness of all ADEs according to the definition given. If the ADE is unexpected (as per the risk analysis or other Reference Safety Information (RSI) document), then it is a USADE.
- Severity
  Assessing the severity of the event according to the following terms and assessments. The intensity of an event should not be confused with the term “serious” which is a regulatory definition based on participant/event endpoint criteria.
    - **Mild**: Some discomfort noted but without disruption of daily life
    - **Moderate**: Discomfort enough to affect/reduce normal activity
    - **Severe**: Complete inability to perform daily activities and lead a normal life

11.2 Reference Safety Information (RSI)

Reference Safety Information (RSI) is the information used for assessing whether an adverse reaction is expected. Section 9 of the Investigator Brochure details information used for assessing whether an adverse reaction is expected in this trial.

11.3 Notification and recording of Adverse Events (AEs), Adverse Device Events (ADEs)

All AE and ADEs are to be documented in the participants' medical notes or other source data documents and the CRF. The period for AE reporting will be from visit one (screening visit) until visit four (day seven). Once assessed, if the AE is not defined as SERIOUS, will not be recorded as an AE on the CRF. AEs must be reported from consent until the participant's last follow-up visit.

11.4 Adverse events that do not require reporting

Refer to section 10.12 for more details on anticipated side effects and their reporting. Any neurological deterioration (including coma), seizures and atrial fibrillation/flutter are common AEs following ischaemic stroke, so will be recorded as predictable outcomes related to the primary presenting pathology.

11.5 Device Adverse Reporting Failures, Malfunctions and Re-use

All device deficiencies will be recorded on the clinical investigation device deficiency log and where appropriate in the participant's medical records. Device deficiencies which could have caused a SADE must be reported to the device manufacturer (Afferent Medical Solution Ltd) and the Sponsor within 24 hours of becoming aware of the event by submitting a Device Reporting Form to a.g@afferentmedicalsolutions.com. Device deficiencies must be recorded and reported throughout the Clinical Investigation. AffeX-CT has been specifically designed to be used by only one patient and must not be re-used by other patients.

11.6 Notification and reporting of Serious Adverse Events (SAEs), and Unexpected Serious Adverse Device Events (USADEs)

Refer to Investigator Brochure sections 9.2 and 9.3. AllSAEs that are considered to be 'related' and 'unexpected' are to be reported to the Sponsor and the Sponsor's representative within 24 hours of learning of the event. The SAEs and reportable device deficiencies must be reported from consent until the participant's last follow-up visit. All Serious Adverse Event (SAEs) and Unexpected Serious Adverse Device Events (USADEs) will be recorded in the participants' notes, the CRF, the Sponsor SAE form and reported to the device manufacturer (Afferent Medical Solution Ltd) and the Sponsor (administered by the Joint Research Management Office or agreed representative) (except those specified in this protocol as not requiring reporting). SAE form and reported to the Sponsor (Joint Research Management Office via mailbox at research.safety@qmul.ac.uk), Afferent Medical Solution Ltd. (a.gourine@afferentmedicalsolutions.com) within 24 hours of the Chief Investigator or Sub-Investigator(s) becoming aware of the event. Nominated Sub-Investigators (as listed on the Investigation site delegation log) will be authorised to sign the SAE forms in the absence of the Chief

Investigator at the participating sites. SAEs and reportable device deficiencies will be reported from consent until the participant's last follow-up visit.

11.7 Sponsor medical assessment

The Sponsor has delegated the responsibility for oversight of investigational device safety profile and medical assessment of safety events to the Chief Investigator as medical assessor The Chief Investigator must review all SAEs and reportable device deficiencies within 72 hours of receipt. This review will encompass seriousness, relatedness, and expectedness. Day 0 for all USADEs is when the USADE is received by the Chief Investigator and / or coordinating team and/ or Sponsor (whichever is first). The CI must also maintain oversite of non-serious AEs reported on the case report forms and review them periodically to confirm agreement. It is expected that the Chief Investigator will achieve oversight of AffeX-CT device safety profile through the trial committees as per section 25.0 of the CIP.

11.8 Procedures for reporting blinded USADEs

The Chief Investigator, as Sponsor medical assessor, will assess the event blinded for all possible active and placebo AffeX-CT device procedures. The device manufacture's safety teams will be unblind and can submit unblind safety reports to the MHRA as required.

11.9 Urgent Safety Measures

The Chief Investigator may take urgent safety measures to ensure the safety and protection of the clinical study participants from any immediate hazard to their health and safety. The measures should be taken immediately. In this instance, the approval of the Competent Authority prior to implementing these safety measures is not required. However, it is the responsibility of the Chief Investigator to attempt, where possible, to discuss the proposed change with the Sponsor and Medical Advisor at the MHRA (via telephone) prior to implementing the change if possible. The Chief Investigator has an obligation to inform both the MHRA and Research Ethics Committee in writing **within 3 days** of implementing the Urgent Safety Measure. They must also submit a substantial amendment documenting the changes with 14 days of implementing the urgent safety measure. The Sponsor must be sent a copy of the correspondence with regards to this matter as soon as it is sent.

12.0 Annual reporting

Annual Progress Report will be written by the Chief Investigator and submitted to the Sponsor for review prior to submission to the REC. The Annual Progress Report is due within 30 days of the anniversary date of the "favourable opinion" letter from the REC.

13.0 Statistical and data analysis

13.1 Sample size calculation

This is a pilot study designed to collect data required to develop a larger efficacy trial. An adjusted sample size of 36 patients is required to have a 90% chance of detecting, as significant at the 5% level, a decrease in the coefficient of variation in systolic blood pressure from 15mmHg in the sham group

to 10mmHg in the VAN group [assuming 5% non-compliance rate in each group]. High blood pressure variability (SBP SD ≥16 mm Hg) is associated with at least 20% worse neurological outcome, as judged by failure to achieve modified Rankin Scale 0–2 90 days after mechanical thrombectomy. Power Analysis & Sample Size software (PASS 2022) was used to determine the sample size. The calculated sample size allows for dropout of 5 patients.

13.2 Learning Curve

There is no learning curve consideration to account for. Training for research staff administering the device will be provided with information about the device and will be supervised on the device usage.

13.3 Planned recruitment rate

The estimated planned recruitment rate is ≥2 participants/week. The mechanical thrombectomy service currently treats >25 patients/month. Duration of participant entry is seven days. The study should be completed within five months, with recruitment carefully monitored by the TMG on a weekly basis. Anticipated screen failure rates are minimal as strict criteria are applied for patients to be eligible for mechanical thrombectomy. From our recent study in acute orthopaedic trauma, the anticipated withdrawal rate is<5%. There are no competing/similar studies recruiting the same cohort of potential participants. The proposed start and end date and duration of funding is realistic to ensure the recruitment targets are feasible.

13.4 End of trial definition

The end of trial is defined as the last trial participant completing their seven day visit. The Chief Investigator is delegated the responsibility of submitting the end of trial notification to REC and MHRA once reviewed by the Sponsor. The end of trial notification must be received by the REC and MHRA within 90 days of the end of the study. If the study is ended prematurely, the Chief Investigator will notify the Sponsor, REC, and MHRA within 15 days, including the reasons for the premature termination.

14.0 Statistical Analysis

A full statistical analysis plan will be developed prior to any safety or final analysis. During the recruitment period the statistician will perform a safety analysis if specifically requested by the Trial Steering Committee (TSC). No safety analysis is planned at the outset. This will be outlined accordingly in the TSC charter.

14.1 Summary of baseline data and flow of participants

Baseline characteristics and clinical data for patients randomised to sham and stimulation will be summarised but not subjected to statistical testing. Numbers (%) for categorical variables and means (SD) or medians (IQR) for continuous variables as appropriate will be provided separately for each group. A log will be kept of eligible patients not recruited to the trial. Reasons for non-participation will be categorised and summarised. Participation in the trial, treatment allocation and completeness of follow-up will be illustrated by a CONSORT flow diagram.

Baseline data will be presented for the following factors, separately by randomised trial group:

- Age
- Sex
- Estimated body-mass index
- Medical comorbidities
- Vital signs

14.2 Analysis of participant populations

The main analyses of the primary and secondary endpoints will be conducted on an intention-to-treat population, consisting of all participants with available data based on the trial group to which they were randomized to, irrespective of their compliance to their prescribed treatment as specified in the protocol. For continuous endpoints, mean, and SD, and number of participants with available data will be presented along with estimated crude and adjusted mean differences between trial arms. Binary endpoints will be shown as the number with each endpoint and total number in each group along with the percentage. For binary endpoints, the crude and adjusted odds ratios (ORs) will be estimated.

14.3 Primary endpoint analysis

The primary outcome - coefficient of variation in systolic blood pressure - will be analysed by repeated measures ANOVA. Significance will be set at $p<0.05$. Similar analyses of secondary outcomes will be undertaken by intention to treat principle, according to the treatment to which they were randomised.

14.4 Secondary endpoint analysis

Blood pressure and heart rate variability measures will be analysed by repeated measures ANOVA (significance set at $p<0.05$), analysed in the same way as for the primary endpoint, as detailed above. Specifically, this entails systolic and diastolic blood pressure variability (standard deviation, average real variability, successive variation and residual standard deviation) plus time and frequency domain measures of autonomic cardiac modulation in the first 24 hours after mechanical thrombectomy. The following secondary outcomes will be reported but not subject to statistical testing.

- Blood pressure variability in the first 24 hours from start of mechanical thrombectomy
- Heart rate variability in the first 24 hours from start of mechanical thrombectomy
- NIH Stroke Scale (NIHSS)
- Arrhythmias
- Myocardial injury

14.5 Safety analysis

Local ear irritation/pain and dermatitis will be recorded. Non-compliance and the reasons for failure to comply will be recorded.

14.6 Procedure(s) to account for missing or spurious data

As the primary outcome uses routinely captured data stored by Barts Health EPR, there is minimal risk of missing blood pressure-related data. Heart rate data will be captured by dedicated Holter monitors

provide by the Chief Investigator. Any missing data will not be inputed. Sensitivity analyses to assess the robustness of study results if there are missing primary outcome data will be undertaken. This pilot study will assess data completeness to inform decisions about missing data for a future main trial. Analysis will use all participants with available endpoint data. The level of missing data will be tabulated for each outcome, presenting the number of subjects with a data record and number with missing data, separately by trial arm. Each endpoint will be analysed on a complete case basis. For continuous variables, distributional assumptions will be hard to assess with the small sample size for this pilot study. However, if any continuous endpoint variables appear to seriously violate distributional assumptions or very extreme outliers observed, then non-parametric alternative approaches to analysis will be undertaken in such cases.

14.7 Other statistical considerations

Any changes to the original analysis plan will be recorded in the SAP (SAP revision history) along with dates, updated SAP version number and description of and reasons for the changes.

15.0 Data handling and record keeping

15.1 Source data and source documents

ISO14155 GCP section 3.47, defines source data as "all information in original records, certified copies of original records of clinical findings, observations, or other activities in a clinical investigation, necessary for the reconstruction and evaluation of the clinical investigation." ISO14155 GCP section 3.48, defines source document as "original or certified copy of printed, optical or electronic document containing source data.” To enable review, monitoring, audit and/or inspection of study source data, the Principal Investigator agrees to keep records of all participating participants. Example of these original documents, and data records include (but are not limited to) electronic participant records/clinical notes, original signed consent form, crib sheets(s), participant trial file, participant diary, participant logbook, recorded data from automated instruments, laboratory notes, questionnaires (paper and/or electronic), evaluation checklists, accountability logs, copies or transcriptions (certified after verification as being accurate and complete) and records kept at the laboratories involved in the trial.

A Source Data Agreement (SDA) will be established by Site team and Sponsor monitoring team detailing a full list of all trial source data documents including what comprises source data, corresponding source data documents and its location. The SDA will be held in the ISF and TMF. All source data will be collated by the Investigation site team.

JRMO Clinical Trial Monitor or delegate will have access to source data and source documents for source data verification aspects of monitoring. Direct access will also be granted to authorised representatives from the Sponsor and the regulatory authorities to permit trial-related monitoring, audits, and inspections.

Study research staff will have access to the patient record to record trial outcomes. Holter data labelled by unique trial IDs will be analysed using Kubios software for offline analysis. Direct access

will be granted to authorised representatives from the Sponsor, host institution, and the regulatory authorities to permit study-related monitoring, audits, and inspections.

15.2 Case Report Forms (CRFs)

Pseudonymised trial data will be captured electronically via electronic case report form (eCRF). The CRF will be designed by the database manager in the Critical Care and Perioperative Medicine Research group with input from the Chief Investigator in accordance to Sponsor requirements. Only trial data including safety events that will be used for statistical analysis will be collected. Chief Investigator, Sub-Investigator(s) and Investigation site team are permitted to record on the eCRF(s). The eCRF will be built and managed on a secure web application.

Direct access to eCRF will be restricted, with only delegated and authorised users will be issued with (trial role related and defined) access to the eCRF. Each user will be assigned specific user roles and rights, and this will be reflected on the respective delegation log.

15.3 Data capture

Source data will be collected by the investigators or the research nurses and captured in an eCRF with electronic signatures and an audit trail. All source data is obtained from site medical notes including the electronic patient record. Direct access to the database will be restricted to named users only. The eCRFs will be completed by the Investigator or suitably trained research staff, as designated in the site delegation log, as accurately and completely as possible throughout the study. Source data will be reviewed as part of the source data verification during site monitoring.

15.4 Transferring and transporting data

All data must be handled in accordance with the Data Protection Act (2018) and General Data Protection Guidelines. No data will be transferred outside of the EEA. Identifiable information will not be stored, transported on any portable device (e.g. laptops, memory sticks, CD /DVDs) unless it is encrypted and will not be sent electronically; if it is not subject to end-to-end encryption. Barts Health Participant Identifiable Data (PID) will not be taken out of Barts Health without participant consent.

15.5 Data Management

Source data verification will be conducted in the eCRF. Final sign-off by the site Principal Investigator will be undertaken. A single final data lock will prevent changes, to protect the final data set. The final dataset will be exported from the study database for analysis by the trial statistician. A trial specific Data Management Plan will be developed by the Coordinating team detailing all key methods of data management for collecting, recording and handling of trial data.

16.0 Confidentiality

The Chief Investigator will be the data custodian for all data generated during the study. The Chief Investigator and the study team will ensure that all participants' identities are protected at every stage of the study. To ensure this, at time of consent each participant will be allocated a unique screening

number by the coordinating team before undergoing any screening procedures. The Principal Investigator is responsible for protecting the identity of participants at their site. Participants will be referred to only by their unique trial identifier whenever data is transferred outside of the site, and in all correspondence between the site and the coordinating centre, co-investigators, Sponsor, or anyone associated with the study. No participants will be individually identifiable from any publications resulting from the study. Information regarding study participants will be kept confidential and managed in accordance with the Data Protection Act (2018), the UK Policy Framework for Health and Social Care Research and Research Ethics Committee approval. All study data will be stored and archived in line with the Medical Device Regulations 2002 and subsequent amendments and the Data Protection Act, as described in JRMO SOP 20 Archiving.

16.1 De-identification of participants

A screening log will be maintained at site throughout the study, detailing the potential participant's initials to allow their identification by relevant site staff. Once the participant has completed screening procedures and is enrolled onto the study, they will be allocated a unique trial identifier by the coordinating team. All data will be de-identified before it is used. Medical record numbers will be the only identifiable information collected from the participants stored within the eCRF to facilitate location of the patient within Barts Health, Royal London Hospital by research staff. Other Identifiable data will be de-identified, kept secure, and maintained by the creation and use of a unique identifier for each participant. Encrypted data will be password protected with limited access afforded to the minimum number of individuals necessary for quality control, audit, and analysis.

17.0 Monitoring, Audit, and Inspection

17.1 Monitoring

The JRMO Clinical Trial Monitor has the responsibility of monitoring the trial. A trial specific Monitoring Plan will be developed by the JRMO at Chief Investigator, detailing all monitoring procedures including onsite visits based on the Sponsor risk assessment; the Sponsor and Chief Investigator will agree on the monitoring plan. The Investigation site team will be initiated and monitored with in line with JRMO SOP 28 – Monitoring.

17.2 Auditing

The Sponsor retains the right to audit any aspect of the study, study sites, or central facilities. In addition, any part of the study may be inspected by the regulatory bodies, and funders where applicable. All sites and vendors are asked to inform the Sponsor if notified of any Audit or inspection affecting this study.

18.0 Compliance

The Chief Investigator will ensure that the protocol and study is conducted in compliance with the principles outlined in the Medical Device Regulations 2002 and subsequent amendments, current UK Policy Framework for Social and health care research (2017), ISO14155 GCP guidelines, the World Medical Association Declaration of Helsinki, the Sponsor's and study specific SOPs, and other regulatory requirements. The study will not commence until approval from the relevant competent

authorities, ethics committees and Sponsor permission to activate sites is received. Any additional terms set by the competent authorities and ethics committees shall be followed. Sites will be individually activated by the Chief Investigator and team; this will not occur until site approval is granted.

18.1 Non-Compliance

Prospective, planned deviations or waivers to the protocol are not allowed under the UK regulations on Clinical Trials and will not be used (i.e., it is not acceptable to enroll a participant if they do not meet the eligibility criteria or restrictions specified in the study protocol). A variety of different sources including monitoring visits, corrective and preventative actions (CAPAs), CRFs, communications and updates will capture non-compliances. Accidental protocol deviations will be documented and reported to the Chief Investigator and Sponsor immediately. The Chief Investigator and the coordinating team will assess non-compliances within 24 hours and inform the Sponsor. Any event deemed by the Chief Investigator to affect participant safety or data integrity will be reported to the Sponsor within 24 hours of the coordinating team becoming aware. Frequent deviations will require immediate action and could potentially be classified as a serious breach. Non-compliance will be captured by CRF documentation. The Sponsor will maintain a log of non-compliances to ascertain if there are any trends developing which need to be escalated.

18.2 Notification of Serious Breaches to GCP and/or the protocol

A 'serious breach' is a breach which is likely to affect to a significant degree:

- The safety or physical or mental integrity of the participants of the study; or
- The scientific value of the study.

The site Principal investigator is responsible for reporting any potential serious breaches to the Sponsor (research.safety@qmul.ac.uk) within **24 hours** of becoming aware of the event. The Chief Investigator is responsible for reporting any potential serious breaches to the JRMO **within 24 hours** of becoming aware of the event. The Sponsor is responsible for determining whether a potential serious breach constitutes a serious breach, and will work with the Chief Investigator to investigate and notify and report to the MHRA and REC (as applicable) within seven working days of becoming aware of the serious breach.

18.3 Amendments to the clinical investigation

Should the Chief Investigator or Sponsor deem it necessary to make an amendment to the Clinical Investigation Plan or to the documents submitted with the Clinical Investigation application, these will be implemented as amendments. The full amendment process is located in JRMO SOP 17.

18.4 Suspension or early termination of the clinical investigation

If the clinical investigation is temporarily suspended, this will be notified to the ethics committee and competent authority via a substantial amendment. A further substantial amendment will be implemented to resume the clinical investigation. If a decision is made to terminate the clinical investigation early the ethics committee and competent authority will be notified within 15 days

through the submission of an End of Study notification form. There are no pre-planned criteria for the suspension or early termination of the investigation.

18.5 Contractual agreements

VANS trial is funded by INNOVATE-UK. Contractual agreements between Sponsor and Afferent Medical Solutions Ltd have been executed, and necessary vendor agreements will be in place before the start of the trial.

19.0 Declaration of interests

The Chief Investigator/ Principal Investigator has stated that he does not have any

- Competing interests.
- Personal or professional relationships with the investigational device manufacturer.
- Ownership interests that may be related to products, services, or interventions considered for use in the study or that may be significantly affected by the study.
- Commercial ties (e.g., pharmaceutical, behavior modification, and/or technology companies).
- Non-commercial potential conflicts (e.g., professional collaborations that may impact on academic promotion).

  Full details will be held within the TMF. Please address enquiries to the Chief Investigator.

The Sponsor requires all study committee members complete competing interest declarations.

20.0 Peer review

The trial design and methodology has undergone independent scientific peer reviews by the funder INNOVATE-UK and an independent suitable experience and independent peer reviewer. This CIP has also been reviewed by the Chief Investigators Institute (William Harvey institute, QMUL) prior to trial sponsorship in principle given by the Sponsor requirements. Evidence of peer reviews will be submitted to REC and HRA.

21.0 Public and Patient Involvement (PPI)

Both the INNOVATE-UK grant application and trial design include review by Public and Patient Involvement (PPI) and the Patient and Public Advisory Group advising Afferent Medical Solutions. The Patient and Public Advisory Group have been involved in the initial design of all trial participant facing literature, usability of the AffeX-CT device prototype and the design of the trial, to ensure that trial proposal is understandable to intended participant population. PPI representatives will also be invited to be members of the Trial Steering Committee.

22.0 Indemnity

The insurance that Queen Mary University of London has in place provides cover for the design and management of the study as well as "No Fault Compensation" for participants, which provides an indemnity to participants for negligent and non-negligent harm. A copy of this document will be filled in the TMF.

23.0 Study committees

The Trial Committees outlined (below) will be established and run in accordance with the Sponsor and Funder requirements.

23.1 Trial Management Group (TMG)

The Trial Management Group (TMG) will be in place for this study; members will meet regularly to discuss all aspects of the trial progression. The terms and conditions of this committee including frequency of meetings is in detail in the TMG charter.

23.2 Trial Steering Committee (TSC)

The TSC will be convened of this study, the role is to provide the overall supervision of the trial. A TSC will review and monitor the progress of the trial, ensuring protocol adherence, appropriate action to safeguard participants is taken and the quality of the trial is maintained. Primarily independent members will form the TSC composition; meetings will occur either face to face or via teleconference and will include an independent chair with at least two other independent members (including an independent Statistician), Participant representative (where deemed necessary), Chief Investigator/sub-Investigator (s), Trial Statistician and Trial Manager, and a non-voting representative from Afferent Medical Solution Ltd and the Sponsor. The terms and conditions of this Committee including the authority and frequency of meetings is in detail in the TSC charter.

23.3 Independent data monitoring committee (IDMC)

The Independent Data Safety Monitoring Committee (IDMC) will monitor participant safety and treatment and review unblinded trial data whilst the trial is ongoing. Members of IDMC will be independent of the Chief Investigator/Sub-Investigator (s), Funders and Sponsors; meetings will occur either face to face or via teleconference and will include three members with at least one Clinician experienced in the clinical area and at least one expert Statistician. Chief Investigator/ Principal Investigator /sub-Investigator(s), Trial Statistician, Trial Manager and/or Afferent Medical Solution Ltd where deemed necessary may receive an invite to the meetings. The terms and conditions of this Committee including the authority and frequency of meetings is in detail in the IDMC charter.

24.0 Publication and dissemination policy

24.1 Publication

Data attained from the entire trial including the sub study or from subsets of the trial will be submitted to peer review journals and published on clinicaltrials.gov website within one year of the End of Trial Definition being met. The Chief Investigator will adhere to the requirements laid out by The International Committee of Medical Journal Editors and is responsible for how the manuscript will be written and edited, including the number and order of authors, the journal to which it will be submitted and all other issues. All publications will be sent to the Sponsor prior to publication and should acknowledge the Sponsor with correct designation “Queen Mary University of London”. It is the Chief Investigator’s responsibility to ensure they meet all requirements laid out by The International Committee of Medical Journal Editors (ICMJE) (http://www.icmje.org/). The clinical

investigation has been registered on ClinicalTrials.gov, the publicly accessible database - NCT05417009. The full study report will be accessible via the public website within one year of the End of the Trial Notification.

24.2 Dissemination policy

The data generated from the entire trial including sub-study will be solely owned by QMUL and upon completion of the trial, the data will be analysed, tabulated and a Final Trial Report prepared. The role of Data Controller will be solely QMUL. The Chief Investigator will be Data Custodian for the entirety of the trial and Afferent Medical Solution Ltd and QMUL serve as Data Processors.

On completion of the trial, the CIP, Final Trial Report, anonymised participant level dataset and/or statistical code for generating the results will be accessible on the clinicaltrials.gov website, and release of this information will require permission from the Chief Investigator and Afferent Medical Solution Ltd. The participating Investigators and employees of QMUL and trial Partners will have rights to publish any of the trial data. There are no time limits or review requirements on the publications, but the funder of the trial is to be acknowledged. Participants will not be notified of the outcome of the trial and may request access to their own trial data from the Chief Investigator after the Final Trial Report has been compiled and results have been published. No professional medical writers will be used.

24.3 Access to the final study dataset

The Chief Investigator, Investigation site team, delegated Coordinating Team and Afferent Medical Solution Ltd will have access to the Final Trial Dataset via the eCRF for analysis and reporting. A full (detailed list) of whom will have access to the Final Trial Dataset will be as per the Data Management Plan and will outline the access, data entry, upload and/or transfer.

25.0 Archiving

During the trial the Chief Investigator is responsible for oversight of all trial documents ensuring secure conditions, safe locations and retention of all records. Archiving of all trial documentation including the eCRF at the End of Trial will be authorised by the Sponsor, following the submission of the End of Trial Report. All essential documents maintained in TMF, ISF, participant files along with the trial dataset will be archived in the QMUL electronic archive for 25 years after the completion of the trial as per Sponsors requirements and Barts Health Policy. Any destruction of essential documents will require authorisation from the Sponsor.

26.0 References

Goyal M, Menon BK, van Zwam WH, Dippel DW, and collaborators. Endovascular thrombectomy after large-vessel ischaemic stroke: a meta-analysis of individual patient data from five randomised trials. *Lancet*. 2016;387:1723-31

Jafari M, Desai A and Damani R. Blood pressure management after mechanical thrombectomy in stroke patients. *J Neurol Sci*. 2020;418:117140.

Tang S, Xiong L, Fan Y, Mok VCT, Wong KS and Leung TW. Stroke Outcome Prediction by Blood Pressure Variability, Heart Rate Variability, and Baroreflex Sensitivity. *Stroke*. 2020;51:1317-1320.

Sykora M, Diedler J, Turcani P, Hacke W and Steiner T. Baroreflex: a new therapeutic target in human stroke? *Stroke*. 2009;40:e678-82.

Ay I, Nasser R, Simon B and Ay H. Transcutaneous Cervical Vagus Nerve Stimulation Ameliorates Acute Ischemic Injury in Rats. *Brain Stimul*. 2016;9:166-73.

Maier B, Turc G, Taylor G, Blanc R, Obadia M, Smajda S, Desilles JP, Redjem H, Ciccio G, Boisseau W, Sabben C, Ben Machaa M, Hamdani M, Leguen M, Gayat E, Blacher J, Lapergue B, Piotin M, Mazighi M and Endovascular Treatment in Ischemic Stroke I. Prognostic Significance of Pulse Pressure Variability During Mechanical Thrombectomy in Acute Ischemic Stroke Patients. *J Am Heart Assoc*. 2018;7:e009378.

van Bilsen M, Patel HC, Bauersachs J, Bohm M, Borggrefe M, Brutsaert D, Coats AJS, de Boer RA, de Keulenaer GW, Filippatos GS, Floras J, Grassi G, Jankowska EA, Kornet L, Lunde IG, Maack C, Mahfoud F, Pollesello P, Ponikowski P, Ruschitzka F, Sabbah HN, Schultz HD, Seferovic P, Slart R, Taggart P, Tocchetti CG, Van Laake LW, Zannad F, Heymans S and Lyon AR. The autonomic nervous system as a therapeutic target in heart failure: a scientific position statement from the Translational Research Committee of the Heart Failure Association of the European Society of Cardiology. *Eur J Heart Fail*. 2017;19:1361-1378.

Roloff EV, Tomiak-Baquero AM, Kasparov S and Paton JF. Parasympathetic innervation of vertebrobasilar arteries: is this a potential clinical target? *J Physiol*. 2016;594:6463-6485.

Stavrakis S, Humphrey MB, Scherlag BJ, Hu Y, Jackman WM, Nakagawa H, Lockwood D, Lazzara R and Po SS. Low-level transcutaneous electrical vagus nerve stimulation suppresses atrial fibrillation. *J Am Coll Cardiol*. 2015;65:867-75.

Mastitskaya S, Marina N, Gourine A, Gilbey MP, Spyer KM, Teschemacher AG, Kasparov S, Trapp S, Ackland GL and Gourine AV. Cardioprotection evoked by remote ischaemic preconditioning is critically dependent on the activity of vagal pre-ganglionic neurones. *Cardiovasc Res*. 2012;95:487-94.

Dawson J, Liu CY, Francisco GE, Cramer SC, Wolf SL, Dixit A, Alexander J, Ali R, Brown BL, Feng W, DeMark L, Hochberg LR, Kautz SA, Majid A, O'Dell MW, Pierce D, Prudente CN, Redgrave J, Turner DL, Engineer ND and Kimberley TJ. Vagus nerve stimulation paired with rehabilitation for upper limb motor function after ischaemic stroke (VNS-REHAB): a randomised, blinded, pivotal, device trial. *Lancet*. 2021;397:1545-1553.

Eva A. Mistry, Tapan Mehta, Akshitkumar Mistry, Niraj Arora, Amy K. Starosciak, Felipe De Los Rios La Rosa, James Ernest Siegler III, Rohan Chitale, Mohammad Anadani, Shadi Yaghi, Pooja Khatri, Adam de Havenon, Blood Pressure Variability and Neurologic Outcome After Endovascular Thrombectomy

A Secondary Analysis of the BEST Study. *Stroke*. 2020; 51:511-518

Appendix A: Assessment of primary and secondary outcomes

Definitions

For the purposes of the trial, the start of mechanical thrombectomy is defined at induction of anaesthesia which is the patient's arrival in the anaesthetic room but before being wheeled into the radiology suite. The end of mechanical thrombectomy is defined as the point the patient leaves the radiology suite to be taken to the post recovery anaesthetic room.

Coefficient of variation of systolic blood pressure measured in each patient during the first 24 hours from the start of mechanical thrombectomy.

This will be collected from routine systolic blood pressure measurements as part of standard care from the start of mechanical thrombectomy for 24 hours. There will be no stipulations on the number of measurements taken. This data will be documented in the patient medical records.

Systolic blood pressure variability from blood pressure measured in each patient during the first 24 hours from the start of mechanical thrombectomy.

Systolic pressure measurements as defined as the primary outcome will be collected as part of standard care from the start of mechanical thrombectomy for 24 hours. The data will be reported as standard deviation, average real variability, successive variation and residual standard deviation. PMID: 31813361

Diastolic blood pressure variability from blood pressure measured in each patient during the first 24 hours from the start of mechanical thrombectomy.

This will be collected from routine diastolic pressure measurements as part of standard care from the start of mechanical thrombectomy for 24 hours. There will be no stipulations on the number of measurements taken. This data will be documented in the patient medical records. The data will be reported as standard deviation, average real variability, successive variation and residual standard deviation.PMID: 31813361

Heart rate variability measured during each period of transcutaneous autonomic neuromodulation.

In addition to routine care all patients will wear a Holter monitor during each of the two intervention periods (for further details please see section 6.7). Heart rate variability will be derived from standard parameters using Kubios software.

NIH Stroke Scale (NIHSS) measured before the start of mechanical thrombectomy and 24 hours after the procedure.

NIHSS will be assessed at the following time points by a member of the research team:

- *any time point after admission to hospital but before induction of anaesthesia*
- *24 hours from start of mechanical thrombectomy*

Clinically recorded arrhythmias within the first 24 hours after the start of mechanical thrombectomy

Defined as electrocardiograph evidence for cardiac rhythm as determined by the treating clinician either documented in the medical records and/ or recorded by a 12 lead ECG.

Absolute levels of serum high-sensitivity troponin-T levels measured before the start of the first transcutaneous autonomic neuromodulation and at the end of the second transcutaneous autonomic neuromodulation.

Additional blood samples to routine care will be collected to measure serum high sensitivity troponin-T (Elecsys, Roche Diagnostics) levels. Blood samples will be collected at any time after hospital admission but before the induction of anaesthesia and within two hours upon the completion of the second transcutaneous autonomic neuromodulation. The time of completion for transcutaneous autonomic neuromodulation is defined as when the device is switched 'on' and 'off' by a member of the research team. If blood samples are not collected, a protocol deviation will be recorded and data point classified as 'missing'.
